# Gain-of-function human *UNC93B1* variants cause systemic lupus erythematosus and chilblain lupus

**DOI:** 10.1084/jem.20232066

**Published:** 2024-06-13

**Authors:** Clémence David, Carlos A. Arango-Franco, Mihaly Badonyi, Julien Fouchet, Gillian I. Rice, Blaise Didry-Barca, Lucie Maisonneuve, Luis Seabra, Robin Kechiche, Cécile Masson, Aurélie Cobat, Laurent Abel, Estelle Talouarn, Vivien Béziat, Caroline Deswarte, Katie Livingstone, Carle Paul, Gulshan Malik, Alison Ross, Jane Adam, Jo Walsh, Sathish Kumar, Damien Bonnet, Christine Bodemer, Brigitte Bader-Meunier, Joseph A. Marsh, Jean-Laurent Casanova, Yanick J. Crow, Bénédicte Manoury, Marie-Louise Frémond, Jonathan Bohlen, Alice Lepelley

**Affiliations:** 1 Laboratory of Neurogenetics and Neuroinflammation, Imagine Institute, INSERM UMR1163, Paris, France; 2 Laboratory of Human Genetics of Infectious Diseases, INSERM UMR1163, Necker Hospital for Sick Children, Paris, France; 3 Department of Microbiology and Parasitology, Group of Primary Immunodeficiencies, School of Medicine, University of Antioquia, Medellín, Colombia; 4 MRC Human Genetics Unit, Institute of Genetics and Cancer, University of Edinburgh, Edinburgh, UK; 5 Faculté de Médecine Necker, Institut Necker Enfants Malades, INSERM U1151-CNRS UMR 8253, Université Paris Cité, Paris, France; 6 Faculty of Biology, Medicine and Health, Division of Evolution and Genomic Sciences, School of Biological Sciences, Manchester Academic Health Science Centre, University of Manchester, Manchester, UK; 7 Department of Paediatric Hematology-Immunology and Rheumatology, Necker-Enfants Malades Hospital, Assistance publique–hôpitaux de Paris (AP-HP), Paris, France; 8 Bioinformatics Core Facility, Université Paris Cité-Structure Fédérative de Recherche Necker, INSERM US24/CNRS UMS3633, Paris, France; 9 St. Giles Laboratory of Human Genetics of Infectious Diseases, Rockefeller Branch, The Rockefeller University, New York, NY, USA; 10 Imagine Institute, Université Paris Cité, Paris, France; 11 Université Toulouse Paul Sabatier, Toulouse, France; 12 Paediatric Rheumatology, Royal Aberdeen Children’s Hospital, Aberdeen, UK; 13 Department of Paediatric Rheumatology, Royal Hospital for Children, Glasgow, UK; 14 Department of Pediatrics, Pediatric Rheumatology, Christian Medical College, Vellore, India; 15 Medical and Surgical Unit of Congenital and Paediatric Cardiology, Reference Centre for Complex Congenital Heart Defects—M3C, University Hospital Necker-Enfants Malades, Paris, France; 16 Université Paris Cité, Paris, France; 17 Department of Dermatology, Hospital Necker-Enfants Malades, AP-HP. Université Paris Cité, Paris, France; 18 Centre for Inflammatory Rheumatism, AutoImmune Diseases and Systemic Interferonopathies in Children (RAISE), Paris, France; 19 Howard Hughes Medical Institute, New York, NY, USA; 20 Department of Pediatrics, Necker Hospital for Sick Children, Paris, France

## Abstract

UNC93B1 is a transmembrane domain protein mediating the signaling of endosomal Toll-like receptors (TLRs). We report five families harboring rare missense substitutions (I317M, G325C, L330R, R466S, and R525P) in UNC93B1 causing systemic lupus erythematosus (SLE) or chilblain lupus (CBL) as either autosomal dominant or autosomal recessive traits. As for a D34A mutation causing murine lupus, we recorded a gain of TLR7 and, to a lesser extent, TLR8 activity with the I317M (in vitro) and G325C (in vitro and ex vivo) variants in the context of SLE. Contrastingly, in three families segregating CBL, the L330R, R466S, and R525P variants were isomorphic with respect to TLR7 activity in vitro and, for R525P, ex vivo. Rather, these variants demonstrated a gain of TLR8 activity. We observed enhanced interaction of the G325C, L330R, and R466S variants with TLR8, but not the R525P substitution, indicating different disease mechanisms. Overall, these observations suggest that UNC93B1 mutations cause monogenic SLE or CBL due to differentially enhanced TLR7 and TLR8 signaling.

## Teaser

David et al. describe UNC93B1 missense substitutions causing systemic lupus erythematosus or chilblain lupus, inherited as either autosomal dominant or autosomal recessive traits. In vitro and ex vivo assays show that these variants confer differential hypermorphic TLR7 and TLR8 signaling.

## Introduction

Systemic lupus erythematosus (SLE) describes a heterogeneous set of clinical phenotypes associated with type I interferon (IFN) upregulation and the presence of autoantibodies targeting nuclear autoantigens (Dorner and Furie, 2019), with chilblain lupus (CBL) classified as a specific cutaneous subtype of SLE (Dubey et al., 2022). The global incidence of SLE has been estimated at 5.14 per 100,000 person-years, with women five times more likely to be affected than men (Barber et al., 2021; Tian et al., 2023). Familial aggregation and higher concordance rates between monozygotic versus dizygotic twins suggest a major hereditary component, with rare monogenic forms of SLE providing important insights into disease pathogenesis (Omarjee et al., 2019; Vinuesa et al., 2023). For example, DNASE1L3 deficiency highlights the role of efferocytosis in lupus pathology (Al-Mayouf et al., 2011; Sisirak et al., 2016), while the relevance of type I IFN signaling to SLE is underlined by the association with the Mendelian type I interferonopathies (Gunther et al., 2015; Lee-Kirsch et al., 2007). B cells are also a key player in lupus causation, with PKCδ deficiency the first described B cell–related form of monogenic lupus (Belot et al., 2013), and heterozygous germline alterations in *IKZF1*, encoding the B cell transcription factor IKAROS, identified as a cause of autoimmunity, including SLE (Hoshino et al., 2017).

Expressed in sentinel cells such as macrophages and dendritic cells (DCs), Toll-like receptors (TLRs) are a family of single-pass membrane-spanning proteins that engage structurally conserved microbial features as part of a coordinated innate and adaptive immune response to pathogens. Endosomal TLR3, TLR7/8, and TLR9 sense nucleic acids, respectively, dsRNA, ssRNA, and CpG DNA. Inherited TLR3 deficiency underlies herpes simplex encephalitis (Zhang et al., 2007) and critical influenza or COVID-19 pneumonia (Zhang et al., 2020), while inherited TLR7 deficiency predisposes to critical COVID-19 pneumonia (Asano et al., 2021). Importantly, TLR7/8 and TLR9 can signal upon sensing both viral- and self-derived nucleic acid (Lind et al., 2022). Reflective of the latter situation, disease in murine models of SLE is attenuated in animals deficient for TLR7 (Marshak-Rothstein, 2006), while SLE-like pathology is driven by TLR7 overexpression (Pisitkun et al., 2006). Further, heterozygous gain-of-function (GOF) variants in human *TLR7* have been shown to cause severe autoimmune phenotypes, specifically lupus and neuromyelitis optica, in humans (Brown et al., 2022; David et al., 2024). In contrast, GOF variants in *TLR8* underlie a different autoinflammatory phenotype, without obvious clinical overlap with SLE (Aluri et al., 2021; Fejtkova et al., 2022). While TLR7 and TLR8 share both agonists and response pathways, TLR7, unlike TLR8, is expressed in plasmacytoid DCs (pDCs), the most potent type I IFN–producing cells. Of note, TLR8 is relatively understudied because of uncertainty over its function in mice.

UNC93B1 (uncoordinated 93 homolog B1) is a highly conserved 597 amino acid 12-pass transmembrane protein expressed in the endoplasmic reticulum (ER). Mouse UNC93B1 is best known for mediating TLR signaling through the trafficking of TLR3, 7, and 9 (Kim et al., 2008; Fukui et al., 2011; Majer et al., 2019a, 2019b). UNC93B1 has also been suggested to act as a chaperone for other ER-resident proteins, including the Ca^2+^ sensor STIM1 (stromal interaction molecule 1) (Maschalidi et al., 2017; Wang and Demaurex, 2022), and the cytosolic DNA signaling adaptor molecule STING (stimulator of IFN genes) (He et al., 2021; Zhu et al., 2022). Mice deficient in UNC93B1 are predisposed to infection and display defective TLR3, 7, and 9 dependent responses (Tabeta et al., 2006), and biallelic loss-of-function *UNC93B1* variants in humans that abolish TLR3, 7, 8, and 9 signaling underlie susceptibility to herpes simplex encephalitis (Casrouge et al., 2006).

The above observations indicate the importance of endosomal TLR signaling and UNC93B1 in immunological homeostasis. In this context, we searched for rare *UNC93B1* variants in patients with SLE or CBL.

## Results and discussion

### Rare missense substitutions in UNC93B1 in probands from five unrelated kindreds

**Figure 1.**
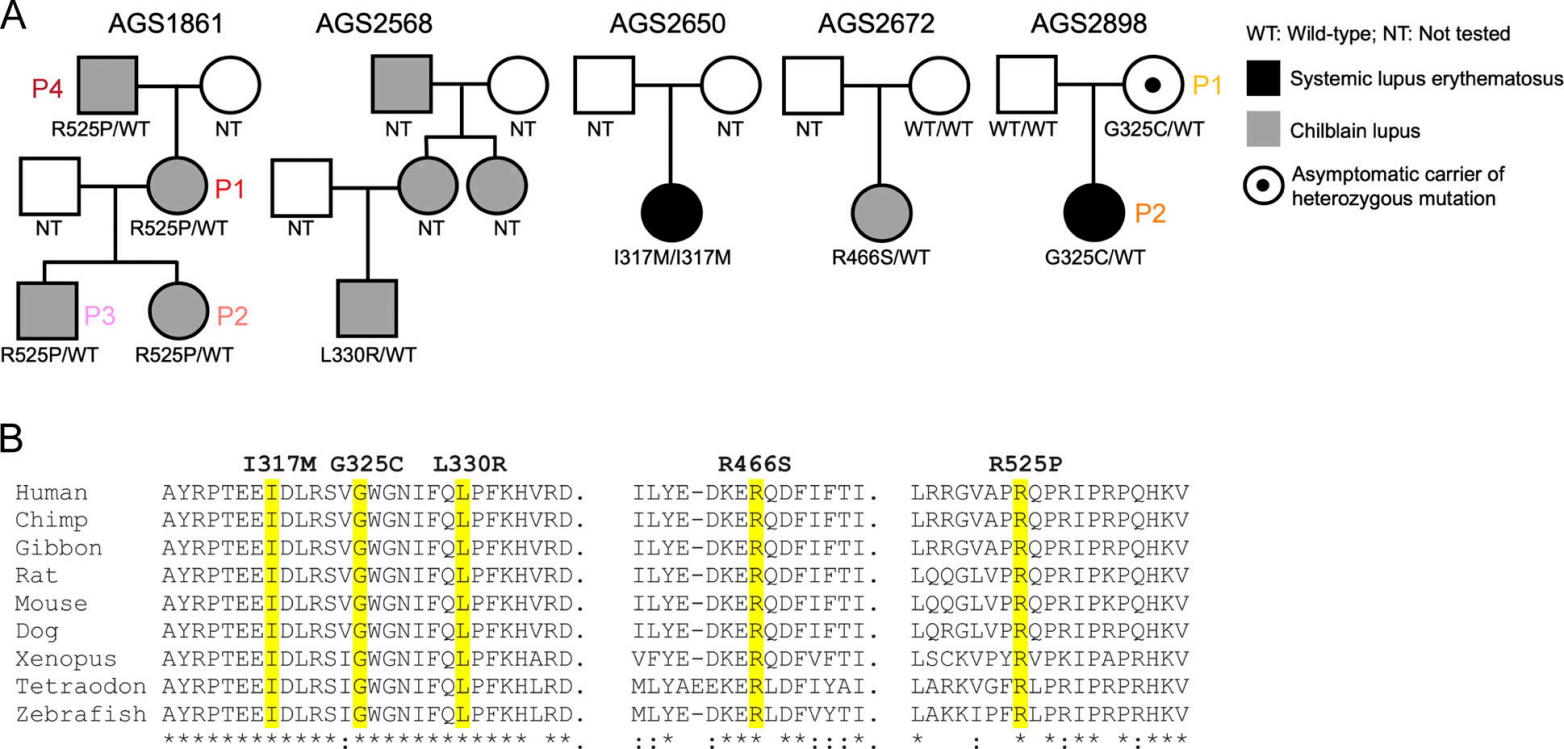
***UNC93B1* genetic data. (A)** Family pedigrees where an affected individual carries a heterozygous or homozygous rare non-synonymous missense substitution in *UNC93B1*. Circles and squares indicate female and male family members, respectively. **(B)** Clustal Omega alignment of UNC93B1 with identified non-synonymous missense substitutions is highlighted in yellow. Alignments are based on the human transcript of UNC93B1: ENST00000227471.7/NM_030930.4; NP_112192.2.

We searched for very rare, that is, a minor allele frequency below 10^−5^, in-frame and out-of-frame variants in protein-coding exons, in the exome sequence data of 63 kindreds (81 patients) with a molecularly uncharacterized diagnosis of either SLE or CBL (39 and 24 kindreds, respectively). In doing so, we identified five probands demonstrating early onset SLE (two probands) or CBL (three probands) with rare non-synonymous missense substitutions in UNC93B1. Four of these probands were heterozygous for a single variant in *UNC93B1* (SLE: p.[Gly325Cys], G325C; CBL: p.[Leu330Arg], L330R; p.[Arg466Ser], R466S; p.[Arg525Pro], R525P). One further patient with SLE was homozygous for a different missense substitution (p.(Ile317Met), I317M) (Fig. 1 A, Table 1, and Table S1).

**Table 1.** Demographic, molecular and clinical data of patients identified to carry rare non-synonymous missense substitutions in *UNC93B1*

Family	Disease segregation	Ethnicity	Nucleotide substitution	Amino acid substitution	gnomAD	Combined VEP damage^a^	CADD	Phenotype
AGS1861	Four (two M, two F) across three generations	North African	c.1574_1575delinsCT (Het)	Arg525Pro (R525P)	0	0.81	-	CBL
AGS2568	M (history of disease in untested mother, MA, and MG)	White European	c.989T>G (Het)	Leu330Arg (L330R)	8/1,552,226	0.93	29.8	CBL
AGS2650	F (parental DNA unavailable)	Indian	c.951C>G (Hom)	Ile317Met (I317M)	0	0.70	24	SLE
AGS2672	F (mother negative, paternal DNA unavailable)	White European	c.1398A>C (Het)	Arg466Ser (R466S)	0	0.86	22.3	CBL
AGS2898	F (variant inherited from asymptomatic mother)	White European	c.973G>T (Het)	Gly325Cys (G325C)	0	0.93	25.7	SLE

CADD, Combined Annotation Dependent Depletion score; F: female; Het: heterozygous; Hom: homozygous; M: male; MA: maternal aunt; MG: maternal grandfather; Pat: paternal. UNC93B1 (ENST00000227471.7/NM_030930.4; NP_112192.2).

a Based on top-performing variant effect predictors from Livesey and Marsh (2023).

The R525P substitution was present in all four affected individuals from a previously described three-generation family with CBL (Beltoise et al., 2018). There was a family history (mother, maternal aunt, and maternal grandmother) of CBL in the proband with the L330R variant, but DNA was unavailable from the other affected individuals. In one case, the variant (G325C) was inherited from a clinically unaffected mother. In a further family, maternal DNA was tested and was negative for the (R466S) variant, but paternal DNA was unavailable. Relating to the proband homozygous for the I317M substitution, neither maternal nor paternal DNA was available.

Four of five missense substitutions were absent from gnomAD, with the L330R variant seen in gnomAD v4 at a frequency of 5 × 10^−6^. Multiple sequence alignment revealed all five substituted amino acid residues to be evolutionarily conserved to zebrafish (Fig. 1 B), with the observed substitutions of these amino acids predicted to be damaging (combined variant effect predictors [VEP] damage score ≥ 0.7) according to a recently published variant effect predictor analysis (Livesey and Marsh, 2023) (Table 1). Using a gene burden test, the cumulative frequency of carriers of non-synonymous *UNC93B1* variants with a minor allele frequency <10^−5^ was 0.4% in gnomAD v4, whereas it was 7.9% in our cohort. This 19-fold enrichment was highly significant at p < 9 × 10^−6^.

Collectively, these findings suggest that the identified *UNC93B1* variants may be disease-causing.

### UNC93B1 substitutions observed in patients with SLE confer enhanced TLR7 signaling

**Figure 2.**
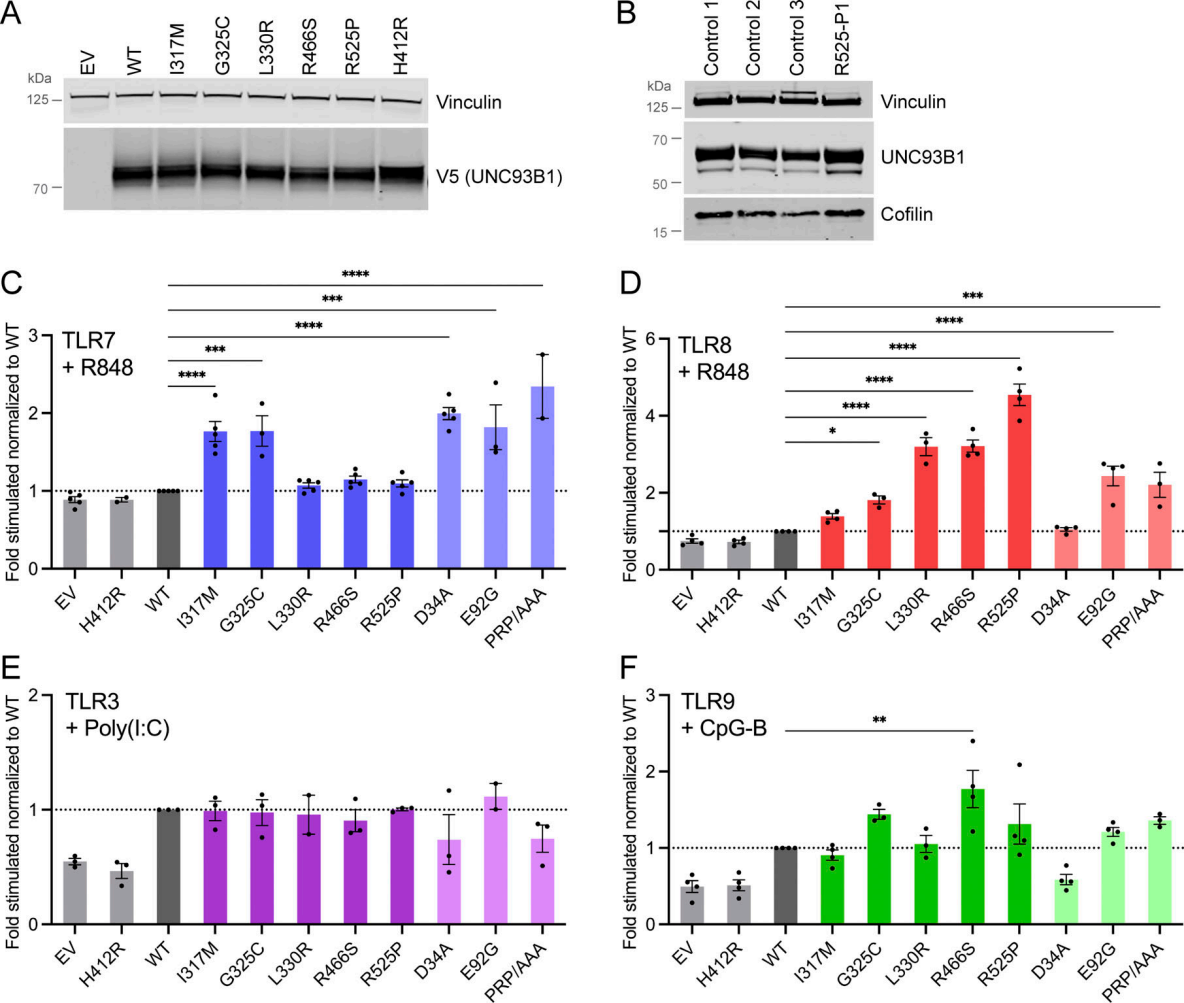
**UNC93B1 variants have different consequences on TLR signaling. (A)** Protein expression level of WT and UNC93B1 variants transfected in HEK293T cells, assessed by western blot using anti-V5 tag for transfected UNC93B1. Vinculin is a loading control. Representative experiment of *n* = 2. **(B)** UNC93B1 protein levels in primary fibroblasts of R525P P1 and three control primary fibroblasts assessed by western blot using indicated antibodies. Vinculin and cofilin are loading controls. Representative experiment. **(C–F)** NF-κB reporter luciferase activity following transfection of HEK293T cells with (C) TLR7, (D) TLR8, (E) TLR3, (F) TLR9 plasmids and EV, WT, and variant UNC93B1 stimulated respectively with (C) R848 0.01 µg/ml, (D) R848 0.1 µg/ml, (E) poly(I:C) 2.5 µg/ml, and (F) CpG-B 1 µM. Data are expressed as the fold-induction of the RLU of the stimulated sample over the RLU of the respective non-stimulated (NS) sample for each UNC93B1 condition (“fold stimulated”), normalized to the fold stimulated obtained for WT UNC93B1. Mean ± SEM of *n* = 3–4 experiments. One-way ANOVA with Dunnett’s post-hoc test: ****P < 0.0001, ***P < 0.001, **P < 0.01, *P < 0.05. Source data are available for this figure: SourceData F2.

**Figure S1.**
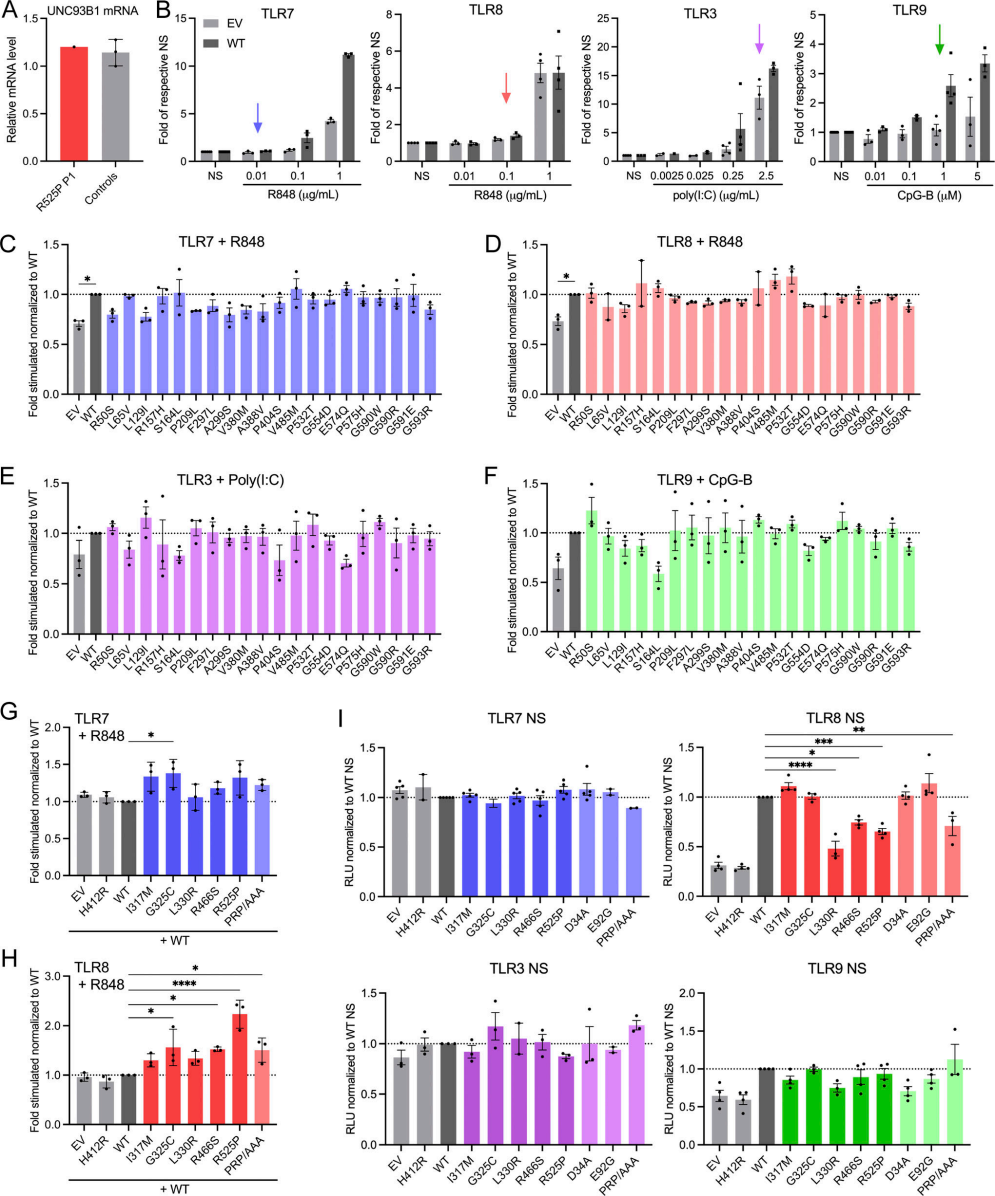
**Effect of patient variants on mRNA expression and of gnomAD variants on TLR signaling. (A)** *UNC93B1* mRNA level in primary fibroblasts of R525P P1 and three control primary fibroblast lines, assessed by qPCR and normalized to *HPRT* mRNA, and expressed as fold over one control fibroblast line dataset. Representative experiment. **(B)** NF-κB reporter luciferase activity following transfection of HEK293T cells with TLR7, TLR8, TLR3, or TLR9 plasmids and EV or WT UNC93B1 stimulated with indicated concentrations of R848, poly(I:C) or CpG-B. Data are expressed as the fold-induction of the RLU of the stimulated sample over the RLU of the respective NS sample for each UNC93B1 condition (“fold of respective NS”). Arrows indicate the dose of ligand chosen to study UNC93B1 variant gain of signaling in Fig. S1, C–F; and Fig. 2, C–F. Mean ± SEM of *n* = 2–4 experiments. **(C–F)** NF-κB reporter luciferase activity following transfection of HEK293T cells with (C) TLR7, (D) TLR8, (E) TLR3, (F) TLR9 plasmids and EV, WT, and gnomAD variant UNC93B1 stimulated respectively with (C) R848 0.01 µg/ml, (D) R848 0.1 µg/ml, (E) poly(I:C) 2.5 µg/ml, and (F) CpG-B 1 µM. Data are expressed as the fold-induction of the RLU of the stimulated sample over the RLU of the respective NS sample for each UNC93B1 condition (“fold stimulated”), normalized to the fold stimulated obtained for WT UNC93B1. Mean ± SEM of *n* = 3 experiments. One-way ANOVA with Dunnett’s post-hoc test. **(G and H)** NF-κB reporter luciferase activity following transfection of HEK293T cells with (G) TLR7 and (H) TLR8 plasmids and WT UNC93B1 together with the same amount of EV, WT and indicated variant UNC93B1 stimulated respectively with (G) R848 0.01 µg/ml, and (H) R848 0.1 µg/ml. Data are expressed as the fold-induction of the RLU of the stimulated sample over the RLU of NS sample for each UNC93B1 condition (“fold stimulated”), normalized to the fold stimulated obtained for WT UNC93B1. Mean ± SEM of *n* = 3 experiments. One-way ANOVA with Dunnett’s post-hoc test. **(I)** NF-κB reporter luciferase activity following transfection of HEK293T cells with TLR7, TLR8, TLR3, or TLR9 plasmids and EV, WT, or variant UNC93B1 without stimulation. Data are expressed as the RLU normalized to WT UNC93B1 RLU. Mean ± SEM of *n* = 2–4 experiments. One-way ANOVA with Dunnett’s post-hoc test. ****P < 0.0001, ***P < 0.001, **P < 0.01, *P < 0.05.

Transfection of the *UNC93B1* missense variants in human embryonic kidney (HEK) 293T cells demonstrated normal protein expression (Fig. 2 A), and testing of fibroblasts from the proband with the R525P substitution (R525P-P1) revealed equivalent expression levels of UNC93B1 protein and mRNA compared with controls (Fig. 2 B and Fig. S1 A). We therefore hypothesized that the *UNC93B1* variants may perturb protein function and not expression. To investigate the effect of the five missense substitutions on TLR signaling, we expressed wild-type (WT) and variant UNC93B1 in HEK293T cells, which lack TLRs, cotransfected with either TLR3, 7, 8, or 9, using an NF-κB luciferase reporter as the read-out. The H412R mutation, also known as the “3d ” mutant, which disrupts mouse TLR3, 7, and 9 signaling (Tabeta et al., 2006), was used as a negative control. We also assessed 20 non-synonymous missense UNC93B1 substitutions identified on gnomAD with a frequency above 1 in 10,000. We measured reporter activity following stimulation with specific TLR agonists (poly(I:C): TLR3; R848: TLR7 and TLR8; CpG-B: TLR9). As highlighted by others (Aluri et al., 2021), responsiveness to ligand stimulation was dose dependent (Fig. S1 B).

All UNC93B1 variants present in gnomAD at a frequency above 1 in 10,000 were isomorphic in their ability to promote TLR3, 7, 8, and 9 signaling, suggesting that UNC93B1 activity with respect to these TLRs is not commonly dysregulated in the general population (Fig. S1, C–F). In contrast, the I317M substitution, seen in the homozygous state in a patient with SLE, conferred a gain of TLR7 activity (Fig. 2 C). This was true to a similar degree for a recently described p.(Glu92Gly) (E92G) homozygous substitution resulting in SLE (Wolf et al., 2024), and a p.(Asp34Ala) (D34A) homozygous substitution causing lupus-like disease in mice through gain of TLR7 function (Fukui et al., 2011). A mutant UNC93B1 (PRP/AAA [position 530–532] in humans, equivalent to PKP/AAA in mouse), leading to enhanced TLR7 signaling due to defective degradation (Majer et al., 2019a), was also associated with enhanced luciferase activity. Notably, of the four heterozygous substitutions seen in our cohort, only the G325C variant (again, observed in the context of early onset SLE), demonstrated an equivalent TLR7-enhancing effect. In contrast, the three heterozygous variants associated with a CBL phenotype were isomorphic with respect to TLR7 activity. The G325C variant also induced a higher NF-κB response when the same amount of WT UNC93B1 was expressed, mimicking the heterozygous state in patients (Fig. S1 G).

Summarizing, *UNC93B1* variants observed in patients with SLE, but not CBL, confer elevated TLR7 signaling in vitro.

### UNC93B1 substitutions observed in patients with CBL confer enhanced TLR8 signaling

Coexpression of our mutant constructs with TLR8, followed by stimulation with R848, demonstrated a gain of TLR8 activity for three (L330R, R466S, R525P; all presenting as CBL) of the four heterozygous variants (Fig. 2 D). While TLR8 activity was also increased with the constructs corresponding to the heterozygous G325C and the homozygous E92G substitutions, as well as the PRP/AAA mutant, this was not as marked. TLR8 activity was not enhanced at all using a D34A construct. These variants showed a similar trend when the same amount of WT UNC93B1 was expressed (Fig. S1 H).

### Normal TLR3 and slightly enhanced TLR9 signaling in vitro

Reporter activity following stimulation with the TLR3 agonist poly(I:C) was essentially equivalent for gnomAD and patient-associated variant constructs, while TLR9 stimulation with CpG-B suggested a slight gain of TLR9 activity for two (G325C, R466S) of the heterozygous variants that we recorded in our cohort (Fig. 2, E and F).

Of note, overexpression of the UNC93B1 variants with TLRs did not result in enhanced NF-κB activity in the absence of stimulation, when compared with WT (Fig. S1 I). The biological relevance of the relative reduction of the signal at baseline for the L330R, R466S, R525P, and PRP/AAA variants when TLR8 is cotransfected is currently unclear.

The above data indicate that all five rare *UNC93B1* variants confer a hypermorphic impact on TLR7 or TLR8 NF-κB signaling, with or without a weak gain of TLR9 signaling, and might act through distinct mechanisms.

### IFN-stimulated gene (ISG) expression and TLR signaling in patient material ex vivo

**Figure 3.**
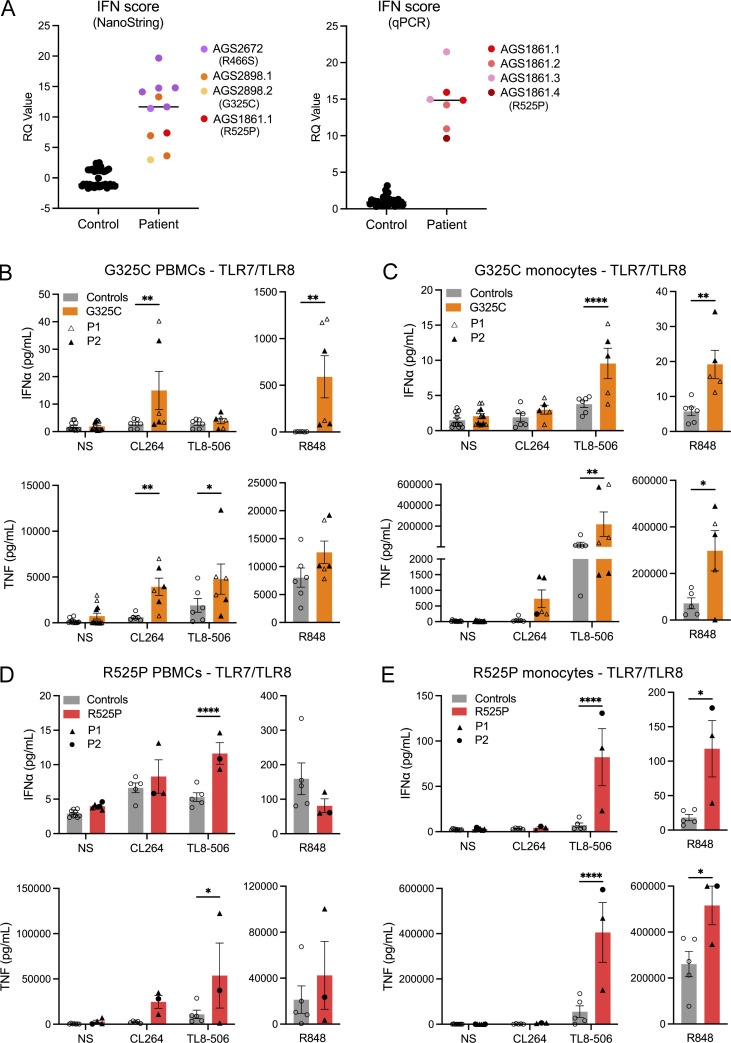
**TLR signaling ex vivo. (A)** ISG expression was measured in controls and patients, using either a 27 (left) or 6 (right) ISG panel (measured on a NanoString platform or using RT-qPCR, respectively) to calculate an IFN score. Colors denote individuals. Values correlate to data presented in Table S1. **(B–E)** Ex vivo stimulation of blood cells. **(B and D)** IFNα and TNF production following stimulation of TLR7 (CL264 5 µg/ml), TLR8 (TL8-506 10 ng/ml), and TLR7/8 (R848 0.5 µg/ml), in bulk PBMCs. **(C and E)** IFNα and TNF production following stimulation of TLR7 (CL264 5 µg/ml), TLR8 (TL8-506 10 ng/ml), and TLR7/8 (R848 0.5 µg/ml) in sorted monocytes. Cells were extracted from healthy individuals (controls), one symptomatic patient (G325C-P2) and one asymptomatic individual (G325C-P1) heterozygous for the G325C substitution (B and C), and two clinically symptomatic patients (R525P-P1 and R525P-P2) heterozygous for the R525P substitution (D and E) in UNC93B1. Mean ± SEM of two to three experiments with individual patient data represented by symbols and pooled according to mutation. Two-way ANOVA with Sidak’s post-hoc test (NS, CL264, and TL8-506) or Mann–Whitney test (R848): ****P < 0.0001, **P < 0.01, *P < 0.05. Source data are available for this figure: SourceData F3.

**Figure S2.**
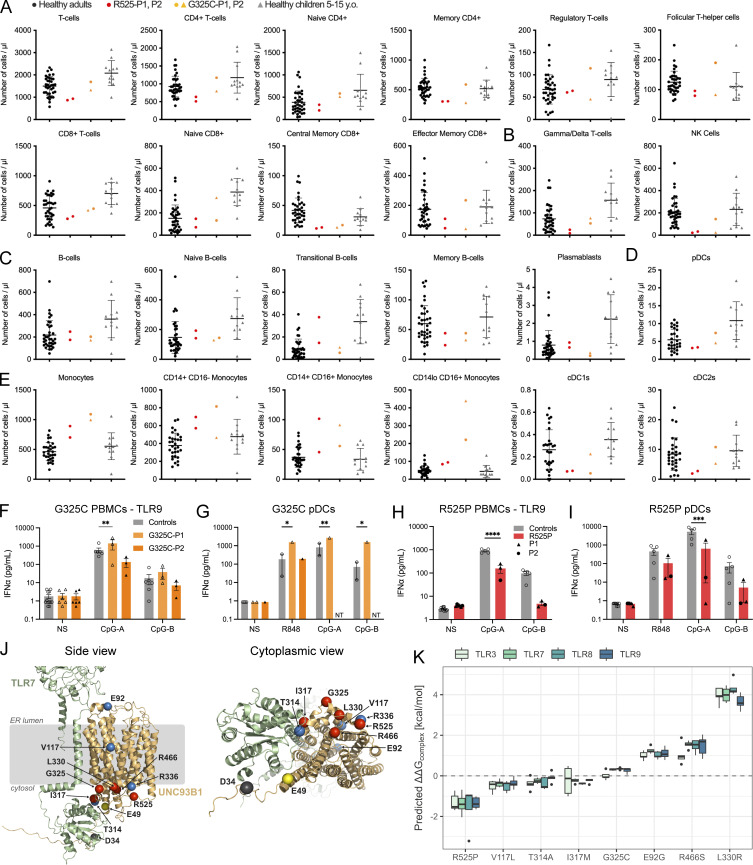
**Immunophenotyping of UNC93B1 patients and structural characterization of variants. (A–E)** Deep immunophenotyping by mass cytometry of immune cell subsets in healthy adults, healthy children 5–15 years old, symptomatic patients carrying the R525P mutation (R525P-P1, R525P-P2), and the asymptomatic mother (G325C-P1) and her symptomatic daughter (G325C-P2) carrying the G325C mutation. **(A)** Immunophenotyping of T cell subsets. **(B)** Immunophenotyping of innate T and lymphoid cell subsets. **(C)** Immunophenotyping of B cell subsets. **(D)** Immunophenotyping of pDCs. **(E)** Immunophenotyping of monocytes and conventional DC subsets. **(F and H)** IFNα and TNF production following stimulation of TLR9 (CpG-A 0.5 µM and CpG-B 0.1 µM) in bulk PBMCs. **(G and I)** IFNα and TNF production following stimulation of TLR7 (R848 0.5 µg/ml, pDCs are not responding to TLR8 ligands) and TLR9 (CpG-B 0.1 µM and CpG-A 0.25 µM [G] or CpG-A 1 µM [I]) in sorted pDCs. NT: not tested. Cells were extracted from healthy individuals (controls), one symptomatic patient (G325C-P2), and one asymptomatic individual (G325C-P1) heterozygous for the G325C substitution (F and G), and two clinically symptomatic patients (R525P-P1 and R525P-P2) heterozygous for the R525P substitution (H and I) in UNC93B1. **(F, H, and I)** Mean ± SEM of two to three experiments with individual patient data represented by symbols and pooled in H and I. **(G)** Single experiment with two controls. Two-way ANOVA with Sidak’s post-hoc test: ****P < 0.0001, ***P < 0.001, **P < 0.01, *P < 0.05. **(J)** Location of pathogenic missense mutations within the structure of the UNC93B1-TLR7 complex (UNC93B1 in yellow; TLR7 in green, AlphaFold-Multimer predicted model). The locations of the five mutations identified in this report are shown in red, together with previously reported D34A mutation (mouse; Fukui et al., 2011) in gray. E92G and R336L (human; Wolf et al., 2024) mutations, and recently published variants at E49 (E49dup; Mishra et al., 2024), V117, and T314 (V117L, T314A; Al-Azab et al., 2023, *Preprint*) are shown in blue. On the right, a view from the cytoplasmic side is represented. **(K)** Predicted structural impact of UNC93B1 variants computed from AlphaFold-Multimer models on UNC93B1 complexes with different TLRs (as shown). Each box contains five data points corresponding to the five AlphaFold models. Box, 25th and 75th percentiles; middle line, median; whiskers, 1.5 times the interquartile range.

In keeping with SLE and CBL, we recorded an upregulation of ISG expression in the whole blood of all seven patients tested, with an abnormal result seen on each of the 18 occasions assessed, including two patients serially assayed three or more times over a period of 1–7 years (Table 1 and Fig. 3 A). Given these data, we hypothesized that altered UNC93B1-dependent TLR activity might be detectable in patient leukocytes, resulting in elevated IFN signaling. To explore this possibility, experiments were conducted using fresh blood (no older than 36 h) collected from the child symptomatic for SLE (G325C-P2) and her clinically asymptomatic mother (G325C-P1) (aged 10 and 42 years, respectively), carrying the G325C variant in UNC93B1, and from the daughter (R525P-P2) and mother (R525P-P1), both symptomatic for CBL (aged, respectively, 20 and 44 years), carrying the UNC93B1 R525P substitution. Deep immune phenotyping, by mass cytometry of peripheral blood mononuclear cells (PBMCs) from these individuals, suggested that leukocyte development and homeostasis were largely unperturbed (Fig. S2, A–E).

We then assessed TLR signaling ex vivo, involving the stimulation of bulk PBMCs, sorted monocytes, and sorted pDCs, and the measurement of cytokine secretion (IFNα and TNF) using a bead-based ELISA system. Consistent with our in vitro data, in the two individuals heterozygous for the G325C substitution, we recorded a gain of TLR7 signaling in bulk PBMCs (containing TLR7-expressing pDCs, DCs, and B cells) stimulated with CL264, and a gain of TLR8 activity in sorted monocytes stimulated with TL8-506, as evidenced by released IFNα and TNF cytokines (Fig. 3, B and C). Consistently, we observed an increased response to R848 (TLR7/8 ligand) in both PBMCs and monocytes. Note that monocytes are essentially unresponsive to TLR7 ligand CL264 stimulation in controls, whereas there was a trend toward a gain of TLR7 signaling in sorted monocytes from G325C patients for TNF (Fig. 3 C). TLR7 stimulation with R848 was also slightly enhanced in the pDCs of the asymptomatic mother (G325C-P1) and comparable with controls in the pDCs of the clinically symptomatic child (G325C-P2) (Fig. S2 G).

Again, concordant with our in vitro data, in the two affected individuals heterozygous for the R525P substitution, we observed a gain of TLR8 activity in monocytes following stimulation with TL8-506 and R848 (Fig. 3 E), as evidenced by elevated IFNα and TNF production. This is consistent with the trend upon TL8-506 stimulation in total PBMCs (Fig. 3 D). The IFN response to TLR7 signaling was similar to controls in bulk PBMCs (Fig. 3 D). In pDCs stimulated with the TLR7 agonist R848, we also observed levels of production of IFNα and TNF comparable with, or slightly lower than, controls (Fig. S2 I).

These ex vivo data indicate enhanced TLR7 and TLR8, or mainly TLR8, activation in patients with *UNC93B1* variants and SLE or CBL, respectively.

Regarding TLR9 responses, we noted a relative gain in TLR9 activity in PBMCs and pDCs from the asymptomatic mother carrying the G325C variant (G325C-P1) (Fig. S2, F and G), consistent with a trend in the reporter assay in 293T cells (Fig. 2 F). However, compared with controls, stimulation of bulk PBMCs with CpG-A/B demonstrated a loss of TLR9 activity in the clinically symptomatic child (G325C-P2) (Fig. S2 F). Consistently, R525P patient pDCs produced lower levels of IFNα upon TLR9 stimulation by CpG-A/B compared with controls, with a similar trend in bulk PBMCs (Fig. S2, H and I). These data suggest a dysregulation of TLR9 signaling in pDCs, possibly consistent with pDC exhaustion (Greene and Zuniga, 2021), and as recorded in a large cohort of patients with SLE (Psarras et al., 2020). Related to this, the normal response to TLR7 ligand stimulation in sorted pDCs from the symptomatic child heterozygous for the G325C substitution may reflect the relative effects of TLR7 gain of signaling vis-a-vis pDC exhaustion related to disease status.

### Modeling of UNC93B1 variants

**Figure 4.**
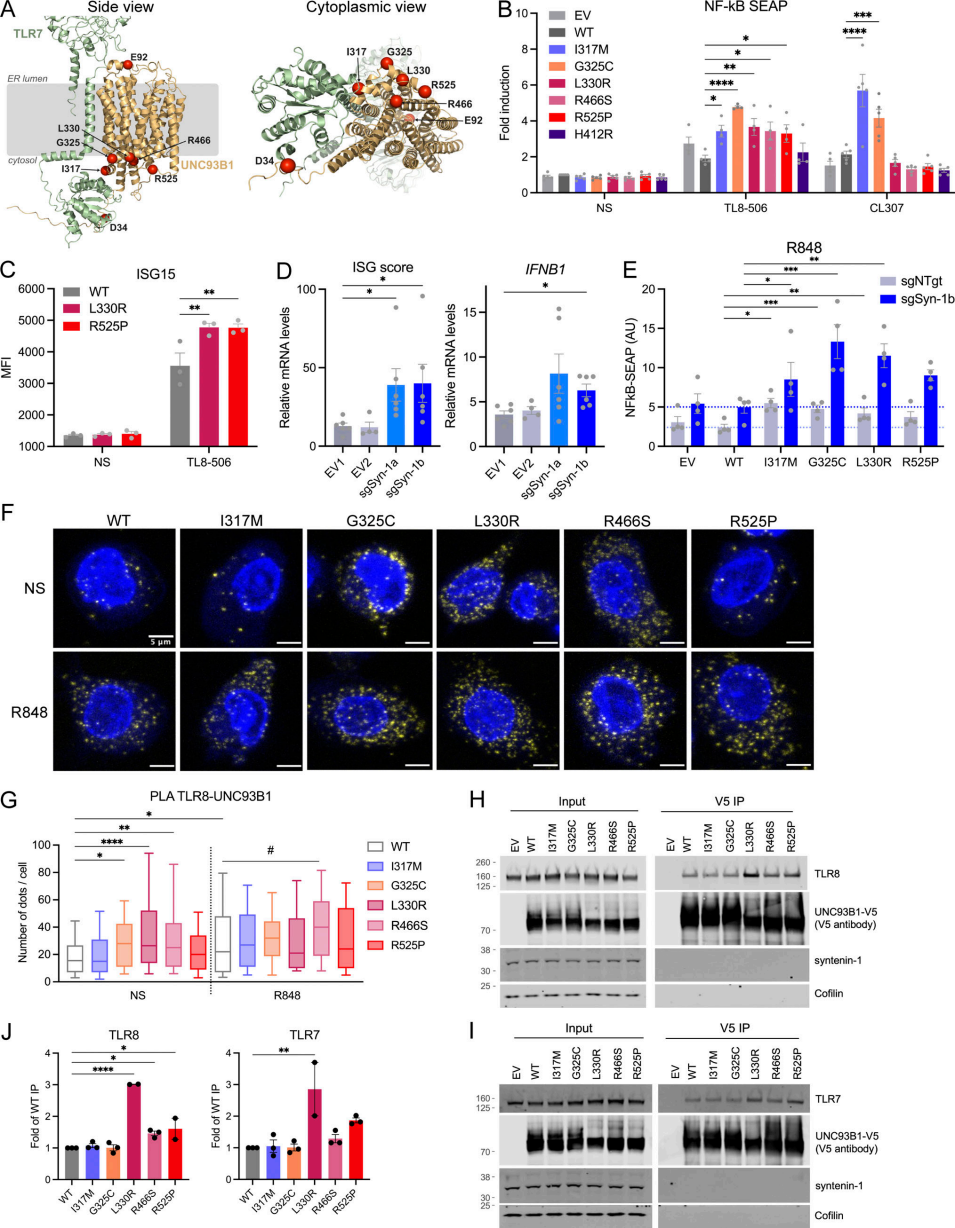
**Interaction of UNC93B1 variants with TLR7/8 and functional consequences. (A)** Location of pathogenic missense mutations within the structure of the TLR7:UNC93B1 complex (AlphaFold-Multimer predicted structure). Note that the TLR7 and TLR8 structures are very similar (TM score of 0.89). The location of the five mutations identified in this report is shown in red, together with the previously described D34A (mouse; Fukui et al., 2011) and E92G (human; Wolf et al., 2024) mutations. On the right, a view from the cytoplasmic side is represented. **(B)** NF-κB SEAP reporter assay in THP-1 Dual cells transduced with EV, WT, and variant UNC93B1 (pTrip-SFFV-GFP-2A construct) unstimulated (NS) or stimulated for 16 h with TL8-506 (0.1 µg/ml) or CL307 (5 µg/ml). Data are expressed as fold induction over WT NS. Mean ± SEM of *n* = 4–5 experiments. Two-way ANOVA with Dunnett’s post-hoc test. **(C)** ISG15 expression, assessed by intracellular staining and flow cytometry, in THP-1 cells stably expressing WT, L330R, or R525P UNC93B1 (pTrip-CMV-Puro-2A construct) and stimulated for 16 h with TL8-506 (1 µg/ml) or NS. Mean ± SEM of *n* = 3 experiments. Two-way ANOVA with Dunnett’s post-hoc test. MFI: mean fluorescence intensity. **(D)** ISG score (median of the relative mRNA levels of the ISGs *IFI27*, *IFI44L*, *OAS1*, and *IFIT1*) and *IFNB1* expression in THP-1 cells stably transduced with EVs (EV1, EV2) or vectors carrying two different sgRNA targeting syntenin-1/*SDCBP* gene (sgSyn-1a and sgSyn-1b) and stimulated for 24 h with TL8-506 (1 µg/ml). mRNA was assessed by qPCR, normalized to *HPRT* mRNA, and expressed as fold induction over EV1 in the NS condition. Mean ± SEM of *n* = 5–6 experiments. Kruskal–Wallis test with Dunnett’s post-hoc analysis. **(E)** NF-κB SEAP reporter assay in control (shNTgt) or syntenin-1 KO (sgSyn-1b) THP-1 Dual cells stably transduced with EV, WT, and variant UNC93B1 (pTrip-SFFV-GFP-2A construct) stimulated for 16 h with R848 (5 µg/ml). Data are expressed as fold induction over WT NS. Mean ± SEM of *n* = 4 experiments. Two-way ANOVA with Dunnett’s post-hoc test. **(F)** PLA showing UNC93B1-TLR8 association (yellow dots) in THP-1 cells stably transduced as in B detected using the Duolink proximity ligation assay with anti-V5 (for UNC93B1) and anti-TLR8 specific antibodies. Cells were treated with R848 (0.5 µg/ml) for 30 min or left untreated (not stimulated, NS). Nuclei (blue) were stained with DAPI. **(G)** PLA signals were quantified with Icy (*n* = 3 experiments, *n* = 104 cells for UNC93B1-WT NS, *n* = 92 cells for UNC93B1-WT + R848, *n* = 113 cells for UNC93B1-I317M NS, *n* = 110 for UNC93B1-I317M + R848, *n* = 97 cells for UNC93B1-G325C NS, *n* = 86 for UNC93B1-G325C + R848, *n* = 118 cells for UNC93B1-L330R NS, *n* = 122 for UNC93B1-L330R + R848, *n* = 99 cells for UNC93B1-R466S NS, *n* = 91 for UNC93B1-R466S + R848, *n* = 107 cells for UNC93B1-R525P NS, *n* = 95 for UNC93B1-R525P + R848) and represented using box (quartiles) and whisker plots with 10–90% error bars. One-way ANOVA with Tukey’s post-hoc analysis for comparisons to WT NS. #: P < 0.05 in WT + R848 versus R466S + R848 comparison. **(H and I)** Co-immunoprecipitation of V5-tagged UNC93B1 in the lysate of 293FT cells cotransfected with TLR8 (H) or TLR7 (I) and UNC93B1-V5 WT and variants using anti-V5 beads (V5 immunoprecipitation), followed by western blot analysis using indicated antibodies (representative blot). **(J)** Quantification of TLR8 or TLR7 band intensity over UNC93B1-V5 band intensity in the IP fraction (related to H and I, respectively). Mean ± SEM of *n* = 2–3 independent experiments. One-way ANOVA with Holm–Sidak’s post-hoc analysis. ****P < 0.0001, ***P < 0.001, **P < 0.01, *P < 0.05. Source data are available for this figure: SourceData F4.

We employed molecular modeling to generate hypotheses to explain the mode of action of our UNC93B1 GOF variants. Modeling of the substituted residues observed in our cohort showed that, like the mutation at D34A that causes murine lupus-like disease in the homozygous state, all five lie on the cytosolic-facing side of the protein (Fig. 4 A and Fig. S2 J). The three heterozygous substitutions at residues 330, 466, and 525, associated with CBL and TLR8 gain-of-signaling—but no in vitro (and, in the case of the R525P substitution, ex vivo) effect on TLR7 activity, map close together in the UNC93B1 protein structure. The isoleucine at 317, with the I317M variant seen in the homozygous state associated with SLE and conferring gain of TLR7 activity in vitro, lies apart from the other substituted residues. The glycine at 325, with the G325C variant seen in the heterozygous state associated with SLE (and clinical non-penetrance in the mother) and conferring gain of TLR7 and TLR8 activity in vitro and ex vivo, is situated between these two groups of residues. Considering the five variants (I317M, G325C, L330R, R466S, R525P), all but L330R are predicted to have a mild structural impact on interactions with TLR7 and TLR8. Of possible note, in contrast to the L330R and R466S substitutions, R525P is predicted to have a stabilizing effect on the complex (Fig. S2 K). The high degree of variant spatial clustering and their moderate predicted structural effect would be consistent with a GOF mechanism (Gerasimavicius et al., 2022).

### Investigation of gain of TLR8 signaling

**Figure S3.**
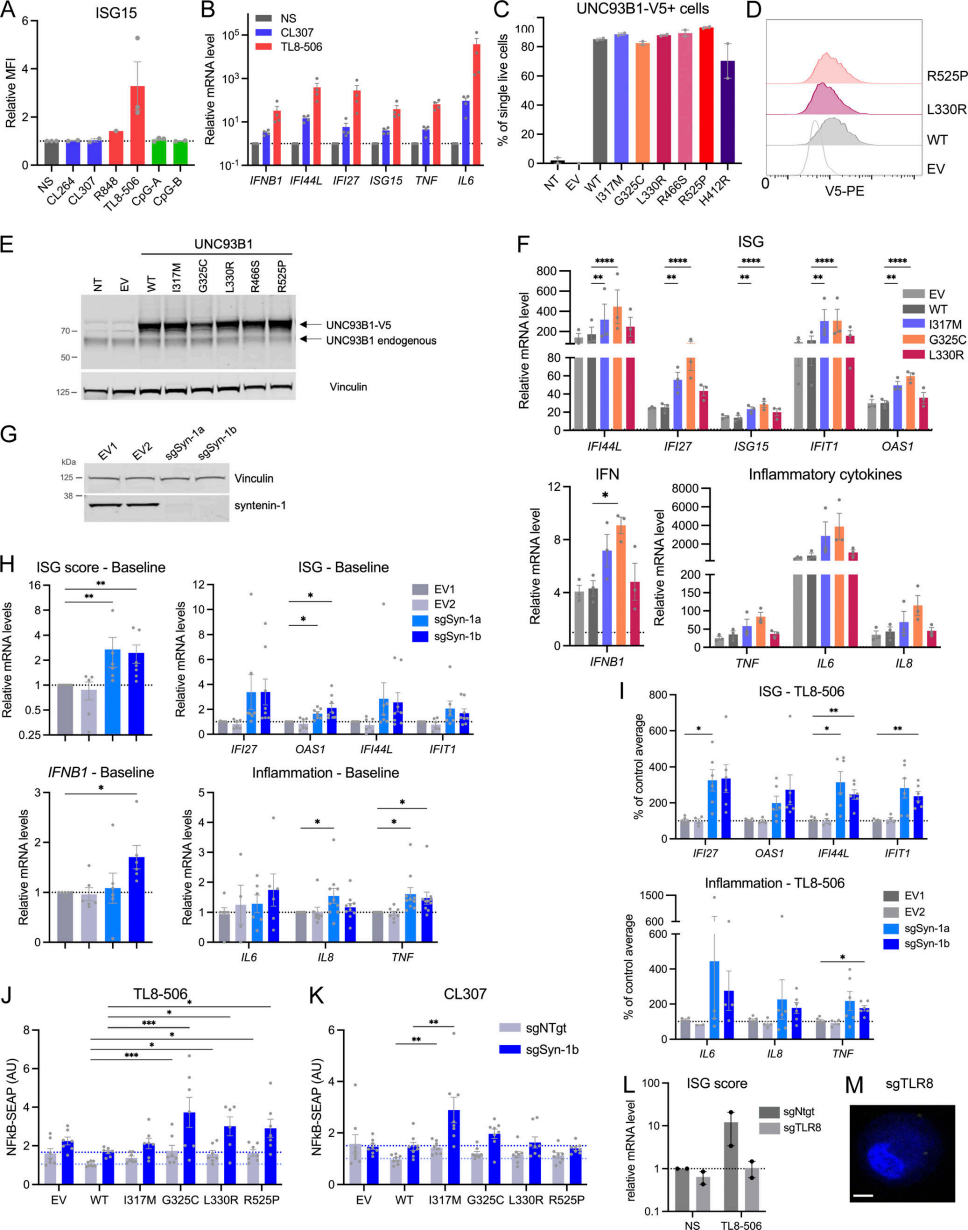
**Characterization of TLR8 signaling in THP-1 cells. (A)** ISG15 expression, assessed by intracellular staining and flow cytometry, in THP-1 cells stimulated for 16 h with CL264 (10 µg/ml), CL307 (TLR7 agonist) (1 µg/ml), R848 (10 µg/ml), TL8-506 (1 µg/ml), CpG-A (5 µM), and CpG-B (5 µM). Relative MFI: mean fluorescence intensity of stimulated condition over NS condition. Mean ± SEM of *n* = 3 experiments. **(B)** Relative mRNA levels of indicated genes in THP-1 cells stimulated for 16 h with CL307 (1 µg/ml) or TL8-506 (1 µg/ml). mRNA was assessed by qPCR, normalized to *HPRT* mRNA, and expressed as a fold induction over NS condition. Mean ± SEM of *n* = 4 experiments. **(C)** V5-tagged UNC93B1 expression, assessed by intracellular staining of V5 and flow cytometry, in THP-1 cells stably expressing EV, WT, and variant UNC93B1 (pTrip-SFFV-GFP-2A construct), or non-transduced (NT). Mean ± SEM of *n* = 3 experiments. **(D)** V5-tagged UNC93B1 expression, assessed by intracellular staining of V5 (PE) and flow cytometry, in THP-1 cells stably expressing WT, L330R, or R525P UNC93B1, or an EV (pTrip-CMV-Puro-2A construct). Overlayed histogram of PE intensity is shown. Representative experiment of *n* = 2. **(E)** Western blot of non-transduced (NT), EV- and UNC93B1-V5-transduced THP-1 protein lysates (pTrip-SFFV-GFP-2A construct), using a conformational anti-UNC93B1 antibodies. Endogenous UNC93B1 migrates at 65 kDa, while the overexpressed, V5-tagged UNC93B1 migrates at a higher molecular weight. **(F)** Relative mRNA levels of indicated genes in THP-1 cells transduced with EV, WT, and variant UNC93B1 (pTrip-SFFV-GFP-2A construct) and stimulated for 16 h with R848 (1 µg/ml), assessed by qPCR, normalized to *HPRT* mRNA, and expressed as fold induction over WT NS condition. Mean ± SEM of *n* = 3 experiments. Two-way ANOVA with Dunnett’s post-hoc test, except for *IFNB1*: one-way ANOVA with Holm–Sidak post-hoc test. **(G)** Syntenin-1 expression level in THP-1 cells stably transduced with EVs (EV1, EV2) or vectors carrying two single sgRNA targeting syntenin-1/*SDCBP* gene (sgSyn-1a and sgSyn-1b), harvested 7 days after transduction and selection, and assessed by western blot. Vinculin is a loading control. Representative experiment of *n* = 3. **(H)** Baseline ISG score (median of the relative mRNA levels of the ISGs *IFI27*, *IFI44L*, *OAS1*, and *IFIT1*), ISGs, *IFNB1*, and inflammatory cytokine (*IL6*, *IL8*, and *TNF*) expression in THP-1 cells stably transduced with EVs (EV1, EV2) or vectors carrying two single sgRNA targeting syntenin-1/*SDCBP* gene (sgSyn-1a and sgSyn-1b), at baseline, harvested 7–10 days after transduction and selection. mRNA was assessed by qPCR, normalized to *HPRT* mRNA, and expressed as a fold induction over EV1. Mean ± SEM of *n* = 6–10 experiments. **(I)** ISGs and inflammatory cytokine (*IL6*, *IL8*, and *TNF*) expression in THP-1 cells stably transduced with EVs (EV1, EV2) or vectors carrying sgRNA targeting syntenin-1/*SDCBP* gene, stimulated for 24 h with TL8-506 (1 µg/ml). mRNA was assessed by qPCR, normalized to *HPRT* mRNA, and expressed as a percentage of the averaged EV1-EV2 fold stimulation over the NS condition. Mean ± SEM of *n* = 6–10 experiments. **(H and I)** Kruskal–Wallis test with Dunnett’s post-hoc analysis (for ISG score and *IFNB1*); mixed-effects analysis (REML; restricted maximum likelihood) with uncorrected Fisher’s test (for ISGs and inflammatory cytokines). **(J and K)** NF-κB SEAP reporter assay in control (sgNTgt) or syntenin-1 KO (sgSyn-1b) THP-1 Dual cell pools stably transduced with EV, WT, and variant UNC93B1 (pTrip-SFFV-GFP-2A construct) stimulated for 16 h with (J) TL8-506 (0.1 µg/ml) and (K) CL307 (5 µg/ml). Data are expressed as fold induction over WT NS. Mean ± SEM of *n* = 7–8 experiments. **(L)** ISG score (median of the relative mRNA levels of the ISGs *IFI27*, *IFI44L*, *OAS1*, and *IFIT1*) in control (sgNTgt) or TLR8 KO (sgTLR8) THP-1 cell pools stimulated for 24 h with TL8-506 (1 µg/ml). mRNA was assessed by qPCR, normalized to *HPRT* mRNA, and expressed as a fold induction over control (sgNtgt) in the NS condition. Mean ± SEM of *n* = 2 experiments. **(M)** PLA assessing UNC93B1-TLR8 association (yellow dots) with anti-V5 (for UNC93B1) and anti-TLR8 specific antibodies, as in Fig. 4 F, in unstimulated TLR8 KO THP-1 cell pools stably transduced with WT UNC93B1-V5. No PLA signal is detected. Nuclei (blue) were stained with DAPI, scale bar: 5 μm. Two-way ANOVA with Dunnett’s post-hoc test. ****P < 0.0001, ***P < 0.001, **P < 0.01, *P < 0.05.

To investigate the effect of mutations on TLR8 signaling, we turned to the monocytic cell line THP-1, which responds with induction of IFN signaling to TLR8 stimulation and minimal IFN signaling to TLR7 and TLR9 stimulation (Fig. S3, A and B). WT and UNC93B1 variants were stably expressed in THP-1 cells by lentiviral transduction (Fig. S3, C–E). Consistent with results derived in HEK293T cells, IFN signaling in response to TLR8 stimulation with TL8-506 was increased for the variants compared with WT, as evidenced by the upregulation of NF-κB signaling using a reporter assay (Fig. 4 B), ISG15 protein (Fig. 4 C), and variably so for the mRNA of ISGs, *IFNB1*, and proinflammatory cytokines (Fig. S3 F). We also identified an increased response to TLR7 ligand CL307 for I317M and G325C variants (Fig. 4 B). The C-terminal tail of UNC93B1 binds the adaptor protein syntenin-1 in mouse macrophages, thereby facilitating the sorting of activated TLR7 into intraluminal vesicles of multivesicular bodies for protein turnover and/or sequestration, with subsequent limitation of signaling in response to self RNA (Majer et al., 2019a). Consistent with this model, a homozygous missense substitution, equivalent to p.(Pro527Thr) in humans, in the C-terminal tail of canine UNC93B1 causes exfoliative cutaneous lupus erythematosus in two dog lines (Leeb et al., 2020). Moreover, in the syntenin-1 model of TLR7 signal limitation, UNC93B1 Lys333 participates in syntenin-1 recruitment through ubiquitination. Since the substituted R525 and L330 residues identified in our cohort lie in these two regions of the protein, and the role of syntenin-1 in TLR8 signaling is unknown, we derived THP-1 cell pools null for syntenin-1 using two different single guide RNAs, and analyzed IFN signaling and proinflammatory cytokine induction at baseline and upon TLR8 stimulation. In doing so, we observed an increased expression of ISGs, *IFNB1*, *IL8*, and *TNF* at baseline in syntenin-1 knock-out (KO) cells (Fig S3 H). We also detected elevated IFN signaling on stimulation with the TLR8 agonist TL8-506, with a similar trend in inflammatory cytokines (Fig. 4 D and Fig. S3 I), suggesting that loss of negative regulation of UNC93B1 by syntenin-1 upregulates not only TLR7 but also TLR8 signaling. However, despite these observations, the GOF conferred by patient-associated mutations was independent of syntenin-1 (Fig. 4 E; and Fig. S3, J and K).

To study a putative gain of UNC93B1-TLR8 interaction, we performed a proximity ligation assay (PLA) between UNC93B1 and TLR8 in THP-1 cells, expressing WT and all five mutant UNC93B1. Supporting a role for UNC93B1 in the regulation of TLR8 signaling, we observed that stimulation of WT UNC93B1 expressing THP-1 cells with the TLR7/8 ligand R848 led to an increase in the UNC93B1-TLR8 interaction (Fig. 4, F and G), while no association was detected in THP-1 TLR8 null cell pools expressing WT UNC93B1 (Fig. S3, L and M). Notably, there was already a marked increase in the association of G325C, L330R, and R466S, but not R525P, with TLR8 at baseline, compared with WT, which was not further enhanced by stimulation, except for R466S. To confirm these results and because of low endogenous TLR8 expression in THP-1 cells, we then performed coimmunoprecipitations in 293FT transfected with tagged UNC93B1 variants and TLR8 or TLR7. In doing so, we observed an interaction between WT and all UNC93B1 variants and TLR8 or TLR7 (Fig. 4, H and I), with a trend toward increased interaction of L330R, R466S, and R525P variants with TLR8 and TLR7 (Fig. 4 J). Combined with the PLA results in THP1 cells, these data suggest different mechanisms involved in GOF of the UNC93B1 variants observed in our patients. Of note, consistent with results in syntenin-1 KO THP-1 cells, syntenin-1 did not coimmunoprecipitate with UNC93B1.

### Concluding remarks

Based on clinical, genetic, and functional in vitro and ex vivo data, we describe five missense substitutions (I317M, G325C, L330R, R466S, and R525P) in UNC93B1 likely causing either SLE or CBL. *UNC93B1* variants explain lupus-related phenotypes in ∼6% (5/63) of kindreds in our cohort, suggesting that UNC93B1 GOF might be one of the most frequent monogenic causes of SLE/CBL. Consistent with a D34A mutation resulting in autosomal recessive lupus-like disease in mice (Fukui et al., 2011) and a homozygous E92G substitution reported in the context of early-onset SLE (Wolf et al., 2024), we observed homozygosity for the I317M substitution in a child with SLE. All four other variants were seen in the heterozygous state, with the G325C substitution, recorded in a child with SLE, inherited from an asymptomatic mother. Our in vitro and ex vivo data indicate a differential effect of distinct mutations on TLR signaling. The D34A mutation in mice has been reported to cause lupus-like disease through enhanced TLR7 activity due to an imbalance of TLR7 and TLR9 trafficking (Fukui et al., 2011) (although it is of note that in our in vitro assay we observed a gain of TLR7 activity in cells that do not express TLR9). We also recorded a gain of TLR7 activity with the I317M (in vitro) and G325C (in vitro and ex vivo) variants, both seen in the context of SLE. In contrast, in the three families segregating CBL, the L330R, R466S, and R525P variants were isomorphic with respect to TLR7 activity in vitro, and for R525P, we showed this to also be the case ex vivo. Rather, these variants conferred gain of TLR8 activity. Consistent with this, we provided evidence to suggest an enhanced interaction of the L330R and R466S variants with TLR8, an effect that is independent of syntenin-1 (even while the latter suppresses TLR8-mediated signaling in WT cells). Interestingly, despite a similar gain of TLR8 signaling, AlphaFold-Multimer modeling indicates that the R525P substitution is predicted to confer a distinct effect on the stabilization of UNC93B1 compared with L330R and R466S. Consistent with a gain in TLR8 signaling, the G325C variant also showed enhanced interaction with TLR8. Like TLR3, TLR7, and TLR9, TLR8 is an endosomal transmembrane protein that signals via UNC93B1, MyD88, and IRAK4 to induce proinflammatory cytokines and type I IFNs. TLR8 is most highly expressed in myeloid cells (neutrophils, monocytes, macrophages, and conventional DCs), and somatic GOF mutations in TLR8 have been reported to cause both immunodeficiency (Aluri et al., 2021) and autoimmune/autoinflammatory disease in males (Fejtkova et al., 2022). All told, these observations likely reflect an underappreciated, carefully regulated role of TLR8 in human immune homeostasis, and crosstalk with other TLRs (de Marcken et al., 2019). The UNC93B1 variants that we identify suggest different mechanisms of gain of TLR7 and TLR8 signaling, providing an opportunity to explore these processes further.

## Materials and methods

### Samples obtained from patients

Samples were obtained from the probands and parents with written informed consent. The study was approved by the Comité de Protection des Personnes (ID-RCB/EUDRACT: 2014-A01017-40) and the Leeds (East) Research Ethics Committee (10/H1307/132).

### Genetic studies

DNA was extracted from whole blood using standard methods. Exome sequencing was performed on genomic DNA using a SureSelect Human All Exon kit (Agilent Technologies) for targeted enrichment and Illumina HiSeq2000 for sequencing. Variants were assessed using the in silico programs SIFT (http://sift.jcvi.org) and Polyphen2 (http://genetics.bwh.harvard.edu/pph2/). Population allele frequencies were obtained from the gnomAD database (http://gnomad.broadinstitute.org). Sanger sequencing was performed to confirm the identified UNC93B1 variants. The reference sequence used for primer design and nucleotide numbering was ENST00000227471.7/NM_030930.4; NP_112192.2.

### IFN status

Whole blood was collected into PAXgene tubes (Qiagen), and total RNA was extracted using a PreAnalytix RNA isolation kit. IFN scores were generated in one of two ways as previously described using TaqMan probes to measure the mRNA expression of six ISGs (*IFI27*, *IFI44L*, *IFIT1*, *ISG15*, *RSAD2*, and *SIGLEC1*) normalized to the expression level of *HPRT1* and *18S rRNA* (Rice et al., 2013). The median fold change of the ISGs is compared with the median of 29 healthy controls to create an IFN score for each individual, with an abnormal score being defined as >2.46. For NanoString ISG analysis, total RNA was similarly extracted from whole blood with a PAXgene (PreAnalytix) RNA isolation kit. Analysis of 24 genes and 3 housekeeping genes (probes of interest [*n* = 24]: *IFI27*, *IFI44L*, *IFIT1*, *ISG15*, *RSAD2*, *SIGLEC1*, *CMPK2*, *DDX60*, *EPSTI1*, *FBXO39*, *HERC5*, *HES4*, *IFI44*, *IFI6*, *IFIH1*, *IRF7*, *LAMP3*, *LY6E*, *MX1*, *NRIR*, *OAS1*, *OASL*, *OTOF*, and *SPATS2L*; reference probes [*n* = 3]: *NRDC*, *OTUD5*, and *TUBB*) was conducted using the NanoString customer designed CodeSets according to the manufacturer’s recommendations (NanoString Technologies). Agilent Tapestation was used to assess the quality of the RNA. 100 ng total RNA was loaded for each sample. Data were processed with nSolver software (NanoString Technologies). The data were normalized relative to the internal positive and negative calibrators, the three reference probes, and the control samples. The median of the 24 probes for each of the 27 healthy control samples was calculated. The mean NanoString score of the 27 healthy controls +2 SD of the mean was calculated. Scores above this value (2.75) were designated as positive.

The “combined VEP damage score” combines predictions from seven top-performing VEPs (DeepSequence, VARITY_R, VARITY_ER, ESM-1v, MetaRNN, Clinpred, and REVEL) as identified in a recent benchmarking study (Livesey and Marsh, 2023). Scores were normalized relative to missense variants observed in UNC93B1 in gnomAD v2.1, with the normalized value representing the fraction of gnomAD variants predicted to be less damaging than the variant of interest and averaged over all seven predictors, as previously described (Robertson et al., 2022).

### Predicting the structural impact of UNC93B1 mutations on TLR interactions

Canonical sequences of TLR3, TLR7, TLR8, and TLR9 (UniProt accession numbers: O15455, Q9NYK1, Q9NR97, and Q9NR96, respectively) and UNC93B1 (Q9H1C4) were retrieved from UniProt (UniProt, 2023). AlphaFold-Multimer (Evans et al., 2022, *Preprint*) predictions were performed with LocalColabFold (https://github.com/YoshitakaMo/localcolabfold), running ColabFold version 1.5.2 (Mirdita et al., 2022) on a single 350 GB NVIDIA A100 GPU. We used templates available in the Protein Data Bank (Berman et al., 2000) and the “mmseqs2_uniref” option for the –msa-mode flag. Five models were generated with three recycles each using the same random seed of 42. The structural impact of UNC93B1 mutations was estimated with FoldX 5.0 (Delgado et al., 2019), by first running the RepairPDB command on all five AlphaFold-Multimer predicted models followed by the BuildModel command to estimate the change in Gibbs free energy upon folding (ΔΔG). The same process was repeated for the monomeric UNC93B1 extracted from the models. The TM (template modeling) score between TLR7 and TLR8 was calculated with TM align (Zhang and Skolnick, 2005) based on the monomers extracted from the top-ranking AlphaFold-Multimer models.

### Plasmids

The pcDNA 3.1 vector encoding V5-tagged human WT UNC93B1 was used as the parental vector for mutagenesis. Mutant plasmids of UNC93B1 were generated via site-directed mutagenesis using the Q5 kit (E0554S; New England Biolabs) according to the manufacturer’s instructions and using oligonucleotides listed in Table S2. NEB 5-alpha competent *Escherichia coli* were transformed with the ligated product and colonies screened for the presence of the desired variants. V5-tagged UNC93B1 WT, L330R, and R525P sequences were then cloned into pTrip-CMV-Puro-2A plasmid (a gift from Nicolas Manel [Institut Curie, France]; plasmid #102611; Addgene; Gentili et al., 2015) using SalI and KpnI sites, and forward primer 5′-ATA​GTC​GAC​ATG​GAG​GCG​GAG​CCG-3′ and reverse primer 5′-ATA​GGT​ACC​TCA​ATG​GTG​ATG​GTG​ATG​ATG-3′. WT and variants V5-tagged UNC93B1 were also cloned into pTrip-SFFV-GFP-2A lentiviral vector plasmid (Gentili et al., 2015) using In-Fusion Snap Assembly (Takara), forward primer GAG 5′-AAC​CCT​GGA​CCT​ATG​GAG​GCG​GAG​CCG​CCG​CTC-3′, and reverse primer 5′-TTT​TCT​AGG​TCT​CGA​TCA​ATG​GTG​ATG​GTG​ATG​AT-3′. TLR3 plasmid was from Invivogen, TLR7, TLR8 (Asano et al., 2021), and TLR9 plasmids have been described previously (Bauer et al., 2001).

### Cell culture and reagents

Human embryonic kidney 293T (HEK293T) and 293FT cells (catalog no. R70007; Invitrogen) were grown in DMEM (GIBCO) supplemented with 10% fetal bovine serum (GIBCO). Primary fibroblasts (three from controls and one from patients R525-P1) were grown in DMEM Glutamax (GIBCO) supplemented with 10% fetal bovine serum and 1% penicillin–streptomycin. THP-1 Dual cells (Invivogen, thpd-nfis) were maintained in RPMI Glutamax (GIBCO) supplemented with 10% fetal bovine serum, 10 mM Hepes, and 0.05 mM 2-mercaptoethanol (GIBCO). All cell lines were cultured at 37°C in 5% CO_2_. All TLR ligands (poly(I:C), CL264, CL307, R848, TL8-506, CpG-A ODN 2216, and CpG-B ODN 2006) and phorbol 12-myristate 13-acetate (PMA) were from Invivogen.

### Luciferase reporter assay

For each condition, 3 × 10^4^ HEK293T cells per well were plated in a 96-well plate in duplicate and transfected with a plasmid containing the Firefly luciferase gene under the control of the human NF-κB promoter (pRL-SV40), a plasmid constitutively expressing Renilla luciferase for normalization (pRL-SV40), as well as a plasmid encoding one of the four TLRs (TLR3, TLR7, TLR8, or TLR9) and a plasmid encoding WT, empty vector (EV), or variant UNC93B1 using the X-tremeGene9 transfection reagent (#6365779001; Sigma-Aldrich) according to the manufacturer’s instructions. After incubation for 24 h, cells were either left unstimulated or stimulated with the corresponding TLR agonist: TLR3-poly(I:C) 2.5 µg/ml for 4 h, TLR7-R848 0.01 µg/ml, TLR8-R848 0.1 µg/ml, or TLR9-CpG-B 1 µM for 24 h (or using concentration indicated in figures). Cells were then lysed, and luciferase levels were measured with the Dual-Luciferase Reporter assay system (#E1980; Promega) according to the manufacturer’s protocol. Luminescence intensity was acquired on an Infinite F200 PRO microplate reader (TECAN). Firefly luciferase activity values were normalized against Renilla luciferase activity to obtain relative luciferase units (RLU) before further data processing (see figure legends).

### Mass cytometry

Whole-blood mass cytometry was performed on 200 μl fresh blood of R525P-P1 and R525P2 of G325C-P1 and G325C-P2 and of controls with a customized antibody panel (Table S3), in accordance with Fluidigm recommendations. Labeled cells were subjected to dead cell staining overnight and then frozen and stored at −80°C until use. The acquisition was performed on a Helios machine (Fluidigm) and the data were analyzed with OMIQ software.

### Ex vivo stimulation of patient PBMCs, monocytes, and pDCs

Whole blood samples (∼50 ml) were collected from patients R525-P1 and R525-P2 of G325C-P1 and G325C-P2 and of healthy individuals into heparin-containing collection tubes. PBMCs were isolated from whole blood by Ficoll-Hypaque density centrifugation no later than 36 h after the blood draw. CD14^+^ cells were isolated from PBMCs by positive selection with human CD14 MicroBeads (#130-050-201; Miltenyi Biotec). pDCs cells were isolated from PBMCs by depletion of non-pDCs using the pDC isolation Kit 2 (#130-097-415; Miltenyi Biotec). After isolation, cells were seeded on a 96-well plate at the confluence of 2 × 10^5^ (PBMCs and monocytes) or 2.10^4^ (pDCs) cells per well. Subsequently, cells were either left unstimulated or were stimulated for 24 h with the corresponding TLR agonist: TLR7-CL264, TLR8-TL8-506, TLR7 and TLR8-R848, TLR9-CpG-A, and TLR9-CpG-B, with concentrations indicated in figure legends. After 24 h, cell supernatants were harvested and the levels of secreted cytokines in the supernatant were determined using the LEGENDplex Human Anti-Virus Response Panel (BioLegend, #740390) according to the manufacturer’s instructions.

### Production of THP-1 cells stably expressing UNC93B1-V5 WT and variants

V5-tagged UNC93B1 WT, L330R, and R525P in pTrip-CMV-Puro-2A, or V5-tagged UNC93B1 WT, I317M, G325C, L330R, R466S, R525P, and H412R in pTrip-SFFV-GFP-2A, lentiviral vector plasmid, were used in combination with packaging vectors psPAX2 (plasmid #12260; Addgene) and envelope pCMV-VSV-G (plasmid #8454; Addgene) to transfect HEK293FT cells using calcium phosphate. Specifically, a medium of 70% confluent 293FT in 75-cm^2^ flasks was changed 2 h before transfection. Calcium phosphate precipitates were prepared by mixing 12.5 µg lentiviral vector with 12.5 µg psPAX2 and 5 µg pCMV-VSV-G plasmids in water for a final volume of 875 µl. 125 µl 2 M CaCl_2_ and 1 ml HBS 2X (50 mM Hepes, 10 mM KCl, 280 mM NaCl, and 1.5 mM Na_2_HPO_4_, pH 7.05) were sequentially added dropwise in a slowly vortexed solution. Solutions were incubated at room temperature for 20 min and mixed gently with 293FT supernatant. The medium was replaced by 8 ml of culture medium 24 h later. After 24 more hours, supernatants were collected, centrifuged at 1,700 rpm for 5 min, and 0.45-µm filtered. 500,000 THP-1 Dual cells were transduced with 0.5 ml lentiviral vectors, 8 µg/ml polybrene (Millipore), and 10 mM Hepes (Invitrogen) in 12-well plates and medium replaced 24 h later. For pTrip-CMV-Puro-2A vector, 1 day after transduction, transduced cells were selected with 1 µg/ml puromycin (Sigma-Aldrich). For pTrip-SFFV-GFP-2A vector, very high levels of V5 expression (Fig. S3C) allowed us to use cells without sorting transduced cells. UNC93B1 expression was verified by western blotting and flow cytometry of V5 staining.

### Production of syntenin-1 KO and TLR8 KO pools in THP-1 cells

Single-guide RNAs (sgRNA) targeting *syntenin-1/SDCBP*, *TLR8*, or non-targeting controls (sgNtgt) (Table S4) were either designed using the CRISPOR tool (http://crispor.tefor.net) (sgSyn-1a), taken from Kashyap et al. (2021) (sgSyn-1b) or taken from Heinz et al. (2020), and cloned into lentiviral construct lentiCRISPRv2-hygro, a gift from Brett Stringer (Flinders University, Adelaide, Australia) (plasmid #98291; Addgene), following the protocol provided on the plasmid website. EV #1 and #2 are two clones obtained after BsmBI digestion to remove buffer sequence, and blunted and ligated using the Quick blunting kit (NEB), following the manufacturer’s instructions. Lentiviral particles were produced and THP-1 Dual cells transduced, as described above for stable expression of UNC93B1. 1 day after transduction, transduced cells were selected with 500 μg/ml hygromycin B (Invivogen), and the selected cell pools were analyzed 7–10 days after transduction. Loss of syntenin-1 expression was verified by western blotting. Loss of TLR8 expression was verified by quantitative PCR (qPCR) after stimulation with TL8-506. Transduced and selected cells were plated in a 24-well plate at 0.5 × 10^6^ cells/ml concentration and stimulated for 24 h with TL8-506 (1 µg/ml), before RNA extraction.

### Western blot analysis

For whole cell lysate analysis, proteins were extracted from THP-1 and primary fibroblasts using radioimmunoprecipitation assay lysis buffer (#89900; Life Technologies) supplemented with 1% protease inhibitor (Halt Protease Inhibitor Cocktail, Life Technologies) and 1% phosphatase inhibitor (Phosphatase Inhibitor Cocktail 2, Sigma-Aldrich), and from HEK293T using digitonin lysis buffer (150 mM NaCl, 50 mM Tris pH 7.4, 5 mM MgCl_2_, 1 % digitonin) with the same inhibitors. Bolt LDS Sample Buffer (4×) (Novex; Life Technologies) and Bolt Sample Reducing agent (10×) (Novex; Life Technologies) were added to protein lysates and denatured at 70°C. Protein extracts were then resolved on 4–12 % Bolt Bis-Tris Plus gels (Invitrogen) and transferred to nitrocellulose membranes (iBlot Invitrogen). Membranes were blocked with 5% non-fat milk in TBS and primary antibodies were incubated overnight in a blocking buffer supplemented with 0.1% Tween. Membranes were washed and incubated with appropriate anti-mouse or anti-rabbit secondary antibodies for 45 min at room temperature (LI-COR System). The signal was detected using the OdysseyCLx System (LI-COR). A list of antibodies used in western blotting is supplied in Table S5.

### qRT-PCR quantification of gene expression

Total RNA was extracted using the RNAqueous-Micro Kit (Ambion), and reverse transcription was performed with the High-Capacity cDNA Reverse Transcription Kit (Applied Biosystems). Levels of cDNA were quantified by qRT-PCR using TaqMan Gene Expression Assays (Applied Biosystems) (Table S6) and normalized to the expression level of *HPRT1*.

### Flow cytometry

For ISG15 expression analysis, WT and variant UNC93B1-V5 (pTrip-CMV-puro-2A construct) transduced and selected THP-1 Dual cells were plated in a 24-well plate at 0.5 × 10^6^ cells/ml concentration and stimulated for 16 h with TL8-506 (1 µg/ml) before fixation. Cells were fixed and stained using the BD Cytofix/Cytoperm Fixation/Permeabilization Kit (#554714). Cells were then stained with PE-anti-ISG15 (#IC8044P; R&D systems) at a 1/100 dilution or rabbit anti-V5 (#13202S; Cell Signalling technologies) at a 1/500 dilution for 30 min at 4°C protected from light. For V5 staining, cells were washed and stained with secondary antibody goat anti-rabbit Alexa Fluor 546 (#A11071; Invitrogen) at a 1/1,000 dilution or goat anti-rabbit Alexa Fluor 647 (#A21244; Invitrogen) at a 1/2,000 dilution. Flow cytometry acquisition was performed on a Novocyte (Agilent) flow cytometer, and results were analyzed using FlowJo software v10.0.

### NF-κB reporter assay in THP-1 cells

WT and variant UNC93B1-V5 (pTrip-SFFV-GFP-2A construct) transduced THP-1 Dual cells were plated in a flat bottom 96-well plate at a density of 10^4^ cells/well/200 µl. Cells were stimulated for 16 h with indicated concentrations of R848, TL8-506, or CL307, and SEAP (secreted embryonic alkaline phosphatase) activity was determined in the supernatant as recommended for the Dual system by Invitrogen. Specifically, 20 µl of supernatant was incubated with 180 µl of QUANTI-Blue solution for 1 h at 37°C before reading the absorbance at 630 nm. Results were normalized to cell viability determined by the CellTiter-Glo Luminescent Cell Viability Assay (G7570; Promega). Both assays were read on a Perkin-Elmer Victor microplate reader.

### Co-immunoprecipitation

293FT cells were cotransfected with either TLR7 or TLR8 plasmids and EV, V5-tagged UNC93B1 WT, or variants in pcDNA 3.1–based plasmids. After 24 h, cells were lysed in co-immunoprecipitation buffer (50 mM Tris pH 7.4, 150 mM NaCl, 0.5% NP-40, 5 mM EDTA) supplemented with 40 mM N-ethylmaleimide (# 04259; Sigma-Aldrich), 1× Halt Protease Inhibitor Cocktail (#78438; Life Technologies), and 1× phosphatase inhibitor Cocktail (# P5726; Sigma-Aldrich). After incubation on ice for 1 h, lysates were cleared by centrifugation. For immunoprecipitation, cleared lysates were incubated overnight at 4°C with V5-Trap Agarose beads (#v5ta; Proteintech), previously washed with a buffer containing 10 mM Tris pH 7.4, 150 mM NaCl, 0.5 mM EDTA, according to the manufacturer’s instructions. After incubation, beads were washed three times in PBS containing 0.5% NP-40. Precipitated proteins were eluted and denatured in 2× SDS loading buffer (2× Bolt LDS Sample Buffer [Novex, Life Technologies], 2.5× Bolt Sample Reducing agent [Novex; Life Technologies] supplemented with 4% SDS [Sigma-Aldrich] and 715 mM 2-Mercaptoethanol [Sigma-Aldrich]) at room temperature for 1 h. Samples were then analyzed by western blotting using TrueBlot: Anti-Rabbit IgG DyLight 800 as a secondary antibody, which preferentially detects native IgG to reveal anti-syntenin-1 immunoblot.

### Proximity ligation assay

WT and variant UNC93B1-V5 (pTrip-SFFV-GFP-2A construct)-transduced THP-1 cells and TLR8 KO THP-1 cell pools were cultured on IBIDI (Clinisciences) with PMA (20 ng/ml) for 72 h. After 48 h, cells were washed and left in complete medium for another 24 h. Cells were stimulated with 0.5 µg/ml of R848 (Invivogen) for 30 min. Cells were then washed, fixed with cold 100% methanol for 3 min at room temperature, and incubated with permeabilization buffer (PBS 0.2% BSA, 0.2% Triton-X100) and primary antibodies (mouse anti-TLR8 antibody, # DDX0480P clone 303F1.14; Novus Biologicals; rabbit anti-V5 antibody, #13202; Cell Signaling). The following steps were carried out using the Duolink PLA reagents (Sigma-Aldrich) according to the manufacturer’s instructions. Images were acquired on a confocal microscope Leica SP8 gSTED and analysis performed using Icy bioimage analysis software.

### Statistics

Statistical analyses were performed with GraphPad PRISM software v10. Values of *n* repeats and statistical parameters for each experiment are reported in the figures and figure legends. Only significant differences are reported in figures unless otherwise indicated.

### Clinical data

#### AGS1861

Previously described in Beltoise et al. (2018), this is a three-generation family of North African ethnicity comprising four affected individuals (a brother and sister aged 22 and 20 years, respectively, their 44-year-old mother [the proband], and their 65-year-old maternal grandfather). Papular, erythematous, purplish, hyperkeratotic, pruritic, and/or painful cutaneous lesions on the hands, elbows, knees, and sometimes the buttocks developed in childhood (6–16 years). These lesions were worse in the winter and improved during the summer. The sister and her mother also demonstrated acrocyanosis, while the grandfather developed linear fibrous lesions on the palmar surface of the proximal interphalangeal joints leading to finger retraction. Additionally, the mother and grandfather reported fragile tooth enamel with numerous cavities and arthralgia. All four individuals used topical steroids and the mother was also treated with hydroxychloroquine. Capillaroscopy and radiography of the hands were unremarkable. Skin biopsy in two patients showed interface dermatitis with vacuolar degeneration of the basal layer and a perivascular lymphocytic infiltrate within the superficial and deep dermis. Direct immunofluorescence showed some IgM and complement deposition along the dermo-epidermal junction. Antinuclear antibodies were raised in three individuals. Complement was normal and there was no renal involvement. A good response to treatment with tofacitinib was seen in the mother.

IFN signaling in blood was elevated in all four patients tested over a period of 4 mo (IFN scores between 9.63 and 21.48 – normal <2.4), and in the proband on one further occasion 5 years later (IFN score 7.37 – normal <2.758). IFN α protein was measured once in the serum of the proband using digital ELISA and found to be raised at 558 fg/ml (normal <10 fg/ml).

All four individuals were heterozygous for a c.1574_1575delinsCT/p.(Arg525Pro) substitution in *UNC93B1* not present on gnomAD.

#### AGS2568

This 10-year-old male was born to non-consanguineous parents of white European ancestry. From the first few years of life, he demonstrated persistent, florid chilblain-like lesions on the fingers and toes with occasional ulceration, also sometimes involving the ears. His mother, maternal aunt, and maternal grandfather were reported to have experienced similar lesions with onset in the first decade of life. Extensive autoantibody screening was negative.

IFN signaling was not assessed.

He was found to carry a heterozygous c.989T>G/p.(Leu330Arg) substitution in *UNC93B1* present on 8/1,552,226 alleles on gnomAD v4. DNA from other family members was unavailable.

#### AGS2650

This 10-year-old female was born to non-consanguineous parents of Indian ancestry. The pregnancy was normal, and she was delivered at term weighing 2.2 kg. She was well until age 18 mo when she developed fever and vomiting and was found to be severely anemic (hemoglobin of 3.4 g/dl). She was diagnosed with autoimmune hemolytic anemia requiring multiple transfusions, steroids, and azathioprine. Antinuclear antibody and double-stranded DNA antibody titers were positive (1:100 dilution) and complement was low (C3 47.6 mg/dl, normal range 90–180; C4 <6.4 mg/dl, normal range 10–40), leading to a diagnosis of SLE. She is developmentally normal. At age 7.5 years her weight was 20 kg (10th centile) and her height was 110 cm (2 SD below the mean).

IFN signaling was not assessed.

She was found to be homozygous for a c.951C>G/p.(Ile317Met) substitution in *UNC93B1* that was not present on gnomAD. Parental DNA was unavailable.

#### AGS2672

This 5-year-old female was born to non-consanguineous parents of white European ancestry. Starting at the age of 4 mo, she developed vasculitic lesions of both the palmar and plantar aspects of the hands and feet and sometimes mouth ulcers. Autoantibodies were consistently negative. Inflammatory markers were modestly but persistently elevated. Skin biopsy at age 2 years showed non-specific inflammatory changes. Her disease has been refractory to treatment with steroids and methotrexate, leading to a trial of baricitinib with some positive benefits.

IFN signaling in blood was elevated on the six occasions tested over a period of >3 years between the ages of 6 and 9 years (IFN scores between 11.41 and 25.92 – normal <2.758).

She was found to carry a heterozygous c.1398A>C/p.(Arg466Ser) in *UNC93B1* that was not present on gnomAD. Her mother does not carry the same variant. Paternal DNA was unavailable.

#### AGS2898

This 10-year-old female was initially diagnosed with pulmonary artery hypertension at age 5 years following two syncopal episodes. Genetic testing was negative, and she was started on nifedipine. Then, aged 7 years, she experienced cutaneous eruptions of the cheeks, associated with cervical and axillary adenopathy, which became more prominent with recurrent episodes of fever. The lesions were not biopsied because of their location, and she was started on tacrolimus. She subsequently also developed a Coombs-positive autoimmune hemolytic anemia, and was positive for antinuclear, anti-RNP, and anti-cardiolipin antibodies, leading to treatment with steroids and mycophenolate in addition to tacrolimus. She is currently in remission.

IFN signaling in blood was elevated on the three occasions tested between the ages of 7 and 10 years (IFN scores of 3.625, 13.295, and 6.93 − normal <2.758).

She was found to carry a heterozygous c.973G>T/p.(Gly325Cys) substitution in *UNC93B1* not present on gnomAD, inherited from her asymptomatic mother.

### Online supplemental material

Fig. S1 shows the effect of patient variants on *UNC93B1* mRNA expression and of UNC93B1 variants on TLR signaling. Fig. S2 describes the results of immunophenotyping of patients identified with UNC93B1 substitutions in this paper and a structural characterization of these and other variants. Fig. S3 describes the characterization of the effect of patient-associated UNC93B1 substitutions on TLR8 signaling in THP-1 cells. Table S1 shows clinical details of patients identified to carry rare non-synonymous missense substitutions in *UNC93B1*. Table S2 shows primers used for site-directed mutagenesis of UNC93B1. Table S3 shows customized antibodies used for mass cytometry on whole blood. Table S4 lists sgRNAs used for the production of syntenin-1 KO and TLR8 KO pools in THP-1 cells. Table S5 lists antibodies used for western blotting. Table S6 lists the primers used for qRT-PCR quantification of gene expression.

## Supplementary Material

Table S1 shows clinical details of patients identified to carry rare non-synonymous missense substitutions in *UNC93B1*.

Table S2 shows primers used for site-directed mutagenesis of UNC93B1.

Table S3 shows customized antibody panel used for mass cytometry on whole blood.

Table S4 shows table of sgRNAs.

Table S5 lists antibodies used in this study for western blotting.

Table S6 lists primers used in this study for qPCR.

SourceData F2 contains original blots for Fig. 2.

SourceData F3 contains original blots for Fig. 3.

SourceData F4 contains original blots for Fig. 4.

## Data Availability

The data in the figures are available in the published article and the online supplemental material.
