# Supplementary material for: Gain-of-function human UNC93B1 variants cause systemic lupus erythematosus and chilblain lupus
Source: J Exp Med. 2024 Jun 13;221(8):e20232066. doi: 10.1084/jem.20232066 (PMC11176256; doi:10.1084/jem.20232066)
Supplement: Table S1 — shows clinical details of patients identified to carry rare non-synonymous missense substitutions in UNC93B1. [file JEM_20232066_TableS1.docx]

**Table S1. Clinical details of patients identified to carry rare non-synonymous missense substitutions in *UNC93B1***

| **Family** | **Mutation** | **Phenotype** | **Age at onset*** | **Features** | **Notable findings** | **Current status and (age*)** | **IFN score** (age in decimalized years)** |
| --- | --- | --- | --- | --- | --- | --- | --- |
| **AGS1861** | R525P | CBL | 6 - 16 | Pruritic/painful cutaneous lesions on hands, elbows, knees, buttocks | Worse in winter, improved in summer | Good response to JAK1/2 inhibition (20 – 65) | *14.96 (16.51),* *21.48 (16.79);* *14.21 (14.48)*, *10.95 (14.77)*; *15.93 (38.44)*, *14.85 (38.73)*; *9.63 (59.63)*; 7.375 (44.32) |
| **AGS2568** | L330R | CBL | <1 | Chilblain-like lesions on fingers, toes and ears with occasional ulceration | AAb testing negative | LTF (10) | Not tested |
| **AGS2650** | I317M | SLE | 1 | AIHA | Positive ANA, anti-dsDNA AAb | LTF (7) | Not tested |
| **AGS2672** | R466S | CBL | <1 | Pruritic/painful cutaneous lesions on hands, feet, mouth ulcers | AAb testing negative | Some response to baricitinib | 14.73 (6.02), 16.71 (6.13), 25.92 (6.81), 17.77 (7.34), 14.15 (8.28), 11.41 (9.74) |
| **AGS2898** | G325C | SLE | 5 | PAH, cutaneous lesions on cheeks, AIHA | Positive ANA and anti-RNP AAb | In remission (10) | 3.625 (7.79), 13.295 (9.32), 6.93 (10.24); 2.975 (42.37) |

AAb: autoantibody; AIHA: autoimmune haemolytic anaemia; IS: immunosuppressant; LTF: lost to follow-up; PAH: pulmonary artery hypertension.

*, years.

**, normal < 2.466 (italicized result); normal < 2.758 (non-italicized results).
