# Supplementary material for: Gain-of-function human UNC93B1 variants cause systemic lupus erythematosus and chilblain lupus
Source: J Exp Med. 2024 Jun 13;221(8):e20232066. doi: 10.1084/jem.20232066 (PMC11176256; doi:10.1084/jem.20232066)
Supplement: Table S2 — shows primers used for site-directed mutagenesis of UNC93B1. [file JEM_20232066_TableS2.docx]

**Table S2. Primers used for site-directed mutagenesis of UNC93B1**

| **Variation(s)** | **Forward primer (5’-3’)** | **Reverse primer (5’-3’)** |
| --- | --- | --- |
| **D34A** | GGCCCCGCTGGCCGAGCTGGTGG | TCGGGCCCGTCCGGGACC |
| **R50S** | AGGAGGAGGAGAGCCGCTACTAC | CCTCGTTGTAGTTGGGGTAC |
| **L65V** | TCAAGAACGTGGTGGCTGCCAGC | GCACGCCCAGGCGCTTGC |
| **E92G** | GCACTACGACGGGACCTACCGCG | AGGATCAGCTGCATCTGCAGGAG |
| **L129I** | ACACACCTGTGATCATCAGGTTTTTTGGAAC | AGAGCAGGGCGGCGATGG |
| **P209L** | GCAGCGGCCTCTGCGGGGCTCCC | TTCATCCCCTGCCCATCCTGCTCCTTGTAGTGG |
| **F297L** | GCAGTGGCCTTGCTGGCCATGCT | CATGAGCACGCTCTCCACCAC |
| **A299S** | TGGCCTTCCTGTCCATGCTGCTG | CTGCCATGAGCACGCTCTC |
| **I317M** | ACGGAGGAGATGGATCTGCGCAGC | GGGCCGGTAAGCGGCTCC |
| **G325C** | GCGTGGGCTGGTGCAACATCTTC | TGCGCAGATCGATCTCCTC |
| **L330R** | CATCTTCCAGCGGCCCTTCAAGCACG | TTGCCCCAGCCCACGCTG |
| **V380M** | CTTACCTCCTCATGGCTTACAGCCTGGG | CCAGCCGCTCCAGCCCCA |
| **A388V** | GGGCGCCTCAGTCGCCTCACTCC | AGGCTGTAAGCCACGAGGAGGTAAGC |
| **H412R** | AGCAGGGGTGCGCCTGCTGCTCAC | CCGGCCACCAGGGGCACC |
| **R466S** | GACAAGGAGAGCCAGGACTTCATC | TTCGTACAAGATTCCCAG |
| **V485M** | CCATCTTCACCATGTACCTGGGC | CCACAGCCTGCCACCAGT |
| **R525P** | GTGGCCCCGCCTCAGCCCCGCATC | GCCCCGGCGCAGCTTCTG |
| **P532T** | GCATCCCGCGGACCCAGCACAAGG | GGGGCTGGCGCGGGGCCA |
| **P575H** | TGGCCCCGAGCACGCTGGACTCG | GGCCTGGGCCCTGCGGGC |
| **G590W** | AACAGGCGCAGAGGGGAGACGGG | CGTACGGGCAGGGCCGGC |
| **G591E** | GGCGCAGGGGGAAGACGGGCCGG | TGTTCGTACGGGCAGGGCCGGC |
| **PRP/AAA** | GGCCCAGCACAAGGTGCGCGGTTACC | GCCGCGATGCGGGGCTGGCGCGG |
