# Supplementary material for: Gain-of-function human UNC93B1 variants cause systemic lupus erythematosus and chilblain lupus
Source: J Exp Med. 2024 Jun 13;221(8):e20232066. doi: 10.1084/jem.20232066 (PMC11176256; doi:10.1084/jem.20232066)
Supplement: Table S3 — shows customized antibody panel used for mass cytometry on whole blood. [file JEM_20232066_TableS3.docx]

**Table S3. Customized antibody panel used for mass cytometry on whole blood**

| **Tag** | **Antibody Panel** | **Clone** | **Catalog #** | **Manufacturer** |
| --- | --- | --- | --- | --- |
| 089Y | CD45 | HI30 | 3089003B | Fluidigm |
| 116Cd | CD66b | QA17A51 | 396902 | Biolegend |
| 141Pr | CCR6 | G034E3 | 3141003A | Fluidigm |
| 142Nd | CD19 | HIB19 | 3142001B | Fluidigm |
| 143Nd | CD127 | A019D5 | 3143012B | Fluidigm |
| 144Nd | CD38 | HIT2 | 3144014B | Fluidigm |
| 145Nd | CD31 | WM59 | 3145004B | Fluidigm |
| 146Nd | IgD | IA6-2 | 3146005B | Fluidigm |
| 147Sm | CD11c | Bu15 | 3147008B | Fluidigm |
| 148Nd | CD20 | 2H7 | 302302 | Biolegend |
| 149Sm | CD25 | 2A3 | 3149010B | Fluidigm |
| 150Nd | NKVFS1 | NKVFS1 | MCA2243GA | Bio Rad |
| 150Nd | KIR3DL1L2 | REA970 | 130-126-489 | Miltenyi Biotec |
| 151Eu | CD123 | 6H6 | 3151001B | Fluidigm |
| 152Sm | TCR-γδ | 11F2 | 3152008B | Fluidigm |
| 153Eu | Va7.2 | 3C10 | 3153024B | Fluidigm |
| 154Sm | CD3 | UCHT1 | 3154003B | Fluidigm |
| 155Gd | CD45RA | HI100 | 3155011B | Fluidigm |
| 156Gd | CCR10 | REA326 | 130-122-317 | Miltenyi Biotec |
| 158Gd | CD27 | L128 | 3158010B | Fluidigm |
| 159Tb | CD1c | L161 | 331502 | Biolegend |
| 160Gd | CD14 | M5E2 | 3160001B | Fluidigm |
| 161Dy | CLEC9A | 8F9 | 3161018B | Fluidigm |
| 162Dy | CD21 | REA940 | 130-124-315 | Miltenyi Biotec |
| 163Dy | CXCR3 | G025H7 | 3163004B | Fluidigm |
| 164Dy | CD161 | HP-3G10 | 3164009B | Fluidigm |
| 165Ho | NKG2C | REA205 | 130-122-278 | Miltenyi Biotec |
| 166Er | CD24 | ML5 | 3166007B | Fluidigm |
| 167Er | CCR7 | G043H7 | 3167009A | Fluidigm |
| 168Er | CD8 | SK1 | 3168002B | Fluidigm |
| 169Tm | NKG2A | Z199 | 3169013B | Fluidigm |
| 170Er | iNKT | 6B11 | 3170015B | Fluidigm |
| 171Yb | CXCR5 | RF8B2 | 3171014B | Fluidigm |
| 172Yb | CD57 | HNK-1 | 359602 | Biolegend |
| 173Yb | HLA-DR | L243 | 3173005B | Fluidigm |
| 174Yb | CD4 | RPA-T4 | 300502 | Biolegend |
| 175Lu | CCR4 | L291H4 | 3175035A | Fluidigm |
| 176Yb | CD56 | NCAM16.2 | 3176008B | Fluidigm |
| 209Bi | CD16 | 3G8 | 3209002B | Fluidigm |
