# Supplementary material for: Gain-of-function human UNC93B1 variants cause systemic lupus erythematosus and chilblain lupus
Source: J Exp Med. 2024 Jun 13;221(8):e20232066. doi: 10.1084/jem.20232066 (PMC11176256; doi:10.1084/jem.20232066)
Supplement: Table S4 — shows table of sgRNAs. [file JEM_20232066_TableS4.docx]

**Table S4. Table of sgRNAs**

| **sgRNA** | **Sense (5’-3’)** | **Antisense (5’-3’)** |
| --- | --- | --- |
| sgNtgt | CACCGATTGGTGCCAATGCTCGGAT | AAACATCCGAGCATTGGCACCAAT |
| sgSyn-1a | CACCGCCTTCAAGTCTTCGAGAGA | AAACTCTCTCGAAGACTTGAAGGC |
| sgSyn-1b | CACCGCTATCCCTCACGATGGAAGT | AAACACTTCCATCGTGAGGGATAGC |
| sgTLR8 | CACCGACAGGAAGTTCCCCAAACGG | AAACCCGTTTGGGGAACTTCCTGTC |
