# Supplementary material for: Gain-of-function human UNC93B1 variants cause systemic lupus erythematosus and chilblain lupus
Source: J Exp Med. 2024 Jun 13;221(8):e20232066. doi: 10.1084/jem.20232066 (PMC11176256; doi:10.1084/jem.20232066)
Supplement: Table S5 — lists antibodies used in this study for western blotting. [file JEM_20232066_TableS5.docx]

**Table S5. List of antibodies used in this study for western blotting**

| **Antibodies** | **Host species** | **Source** | **Reference** |
| --- | --- | --- | --- |
| Cofilin | Rabbit monoclonal | Cell Signaling | 5175 |
| Vinculin | Mouse monoclonal | Santa Cruz | sc-73614 |
| Syntenin-1/MDA9 (E2I9L) | Rabbit monoclonal | Cell Signaling | 27964 |
| UNC93B1 | Rabbit polyclonal | Atlas Antibodies | HPA038716 |
| UNC93B1 (conformational antibody) | Rabbit polyclonal | Melanie Brinkmann | Brinkmann et al., 2007 |
| V5-Tag (D3H8Q) | Rabbit monoclonal | Cell Signaling | 13202 |
| TLR7 | Rabbit monoclonal | Cell Signaling | 5632 |
| TLR8 | Mouse monoclonal | Novus Biologicals | DDX0480P |
| IRDye 800CW (secondary antibody) | Goat anti-rabbit | Li-COR | 926-32211 |
| IRDye 640RD (secondary antibody) | Goat anti-mouse | Li-COR | 926-68070 |
| TrueBlot®: Anti-Rabbit IgG DyLight™ 800 (secondary antibody for syntenin-1 in coimmunoprecipitation WB) | Mouse anti-rabbit | Rockland Immunochemicals Inc | 18-4516-32 |
