# Supplementary material for: Gain-of-function human UNC93B1 variants cause systemic lupus erythematosus and chilblain lupus
Source: J Exp Med. 2024 Jun 13;221(8):e20232066. doi: 10.1084/jem.20232066 (PMC11176256; doi:10.1084/jem.20232066)
Supplement: Table S6 — lists primers used in this study for qPCR. [file JEM_20232066_TableS6.docx]

**Table S6: List of primers used in this study for qPCR**

| **Primers** | **Sequence** |
| --- | --- |
| *HPRT1* | Hs03929096_g1 |
| *IFI27* | Hs01086370_m1 |
| *IFI44L* | Hs00199115_m1 |
| *ISG15* | Hs00192713_m1 |
| *RSAD2* | Hs01057264_m1 |
| *UNC93B1* | Hs00276771_m1 |
| *IFNB1* | Hs01077958_s1 |
| *TNF* | Hs01113624_g1 |
| *IL6* | hs00985639_m1 |
| *IFIT1* | hs00356631_g1 |
| *OAS1* | Hs00973637_m1 |
| *IL8* | Hs00174103_m1 |
