# Supplementary figures and images for: Gain-of-function human UNC93B1 variants cause systemic lupus erythematosus and chilblain lupus
Source: J Exp Med. 2024 Jun 13;221(8):e20232066. doi: 10.1084/jem.20232066 (PMC11176256; doi:10.1084/jem.20232066)

Figure 2A

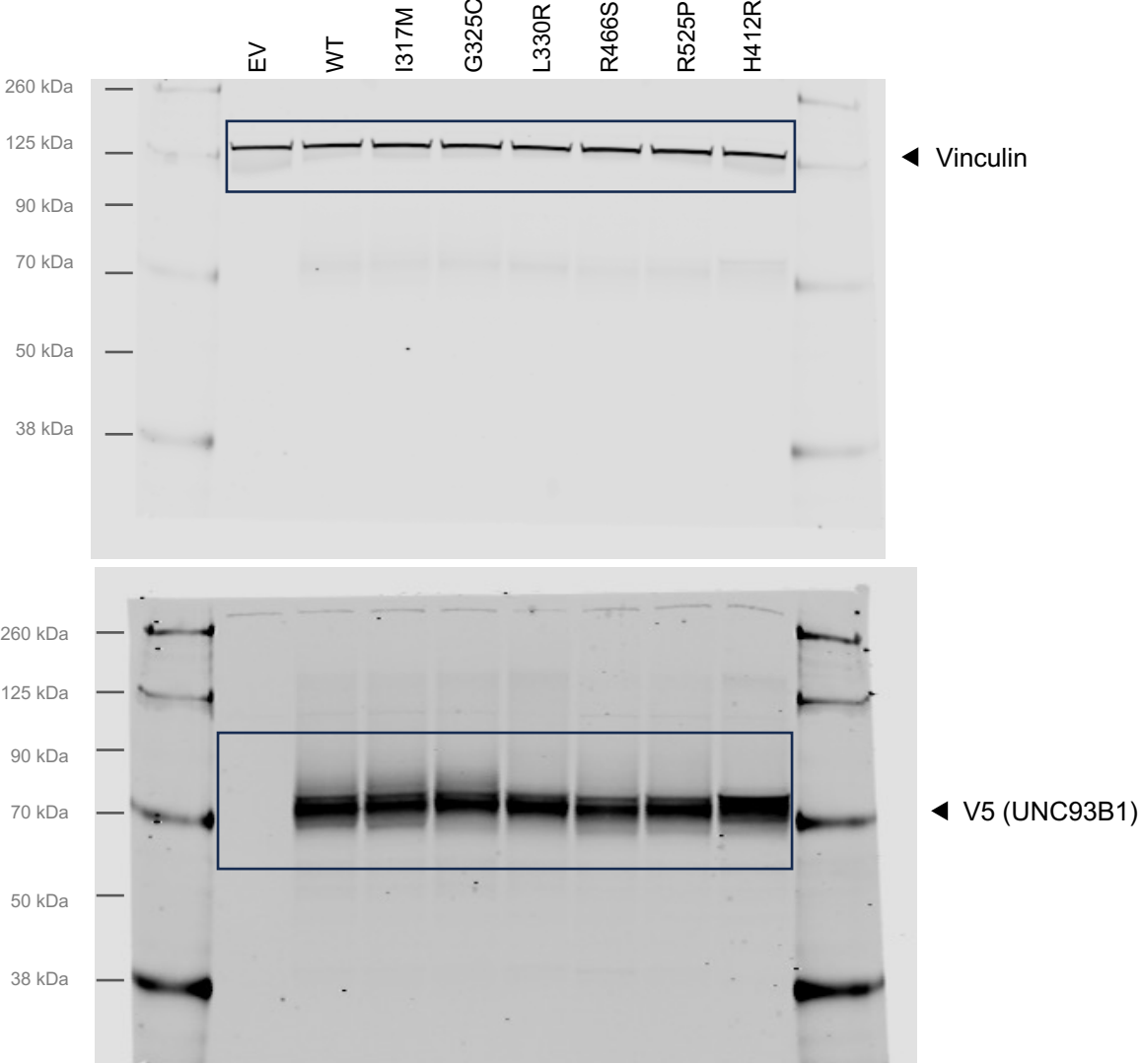

Figure 2B

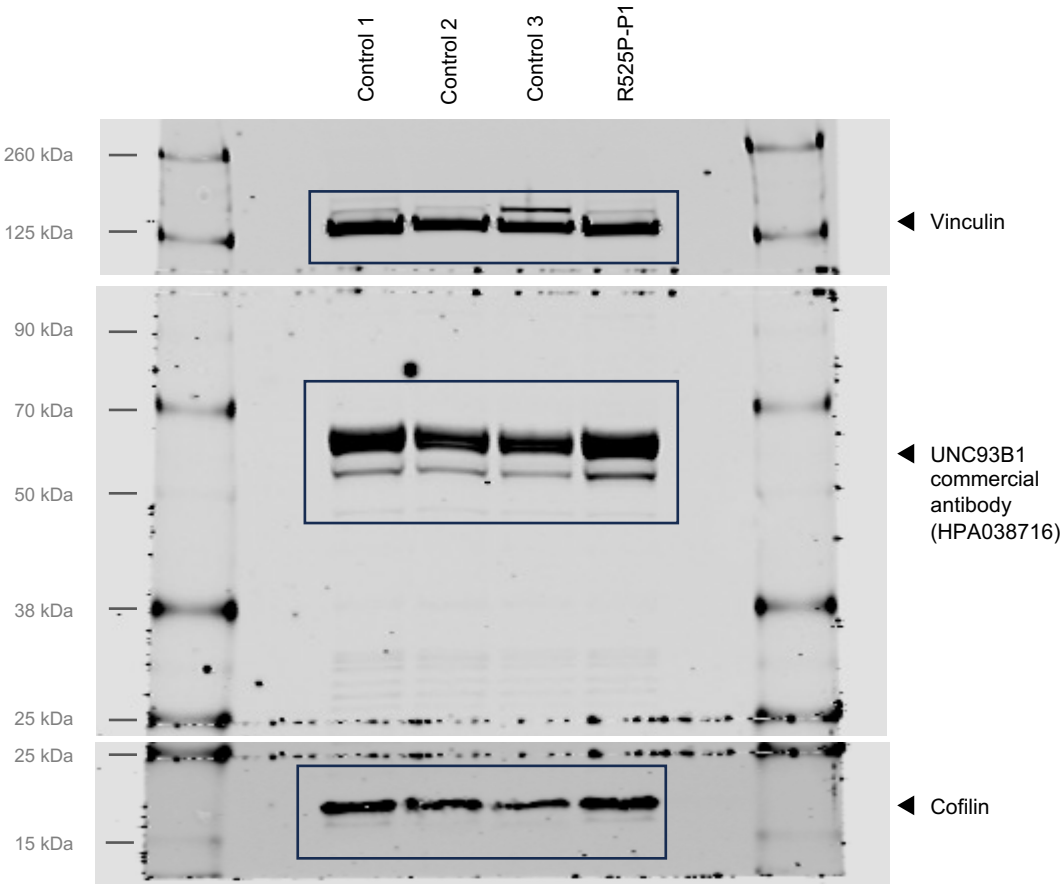

Supplement: SourceData F2 — contains original blots for Fig. 2. [file JEM_20232066_SourceDataF2.pdf]

Figure S3E

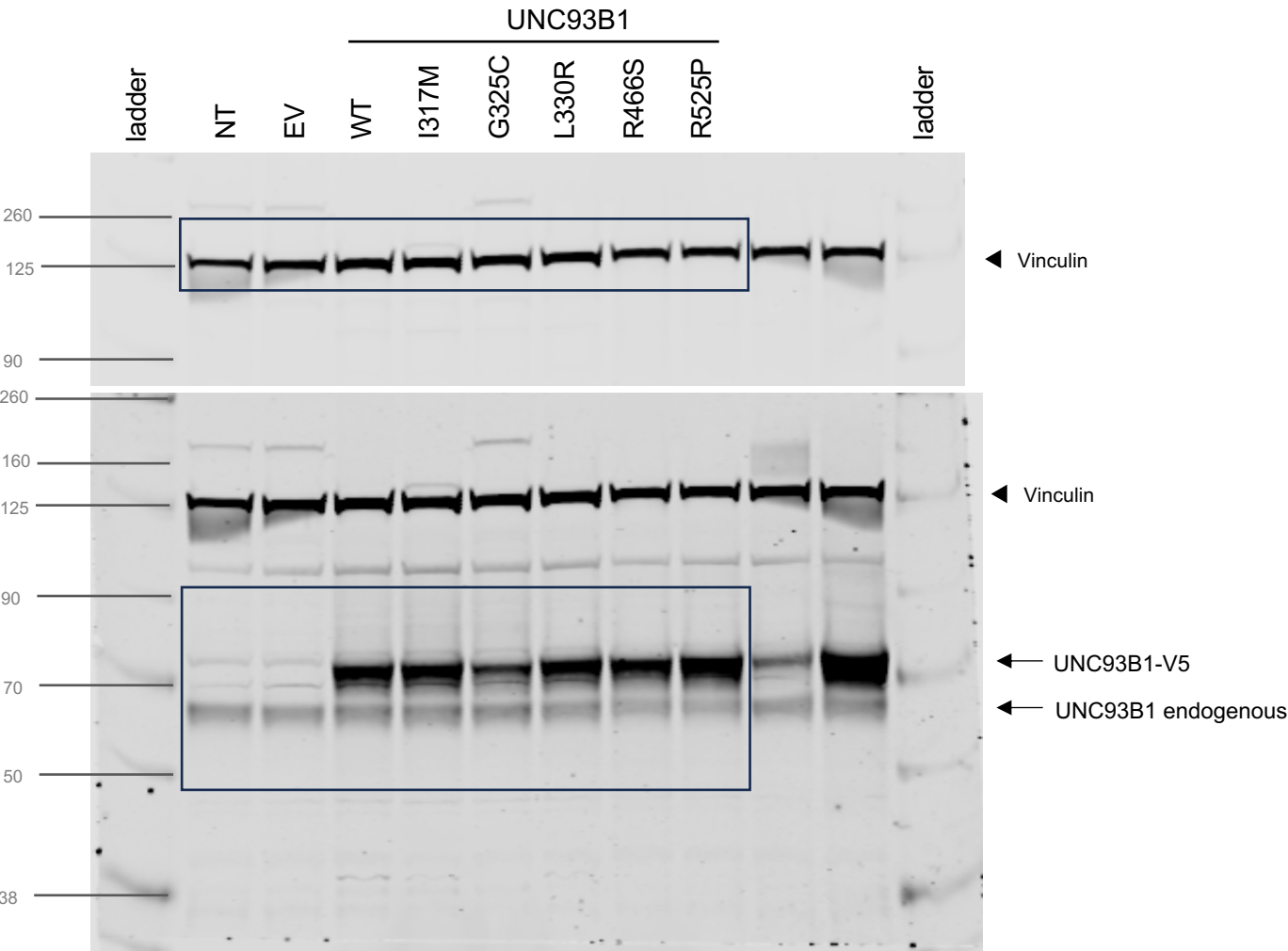

Figure S3G

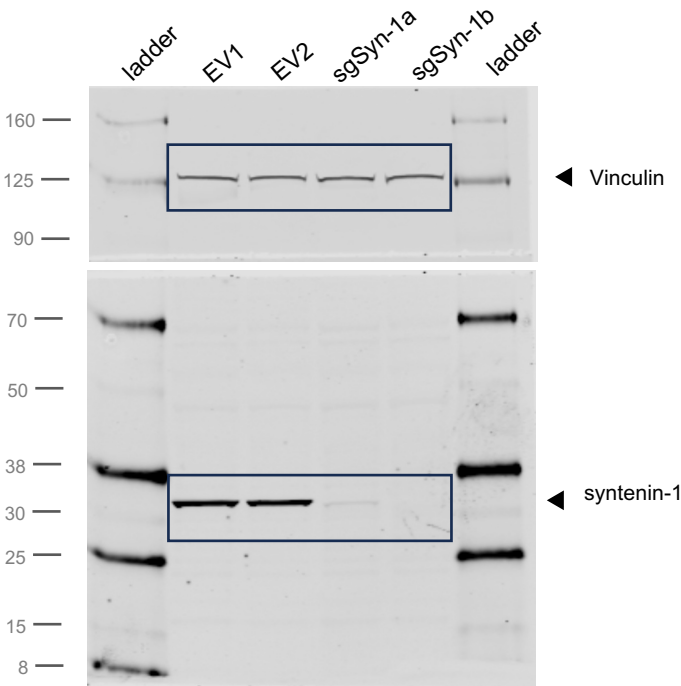

Supplement: SourceData F3 — contains original blots for Fig. 3. [file JEM_20232066_SourceDataF3.pdf]

Figure 4H

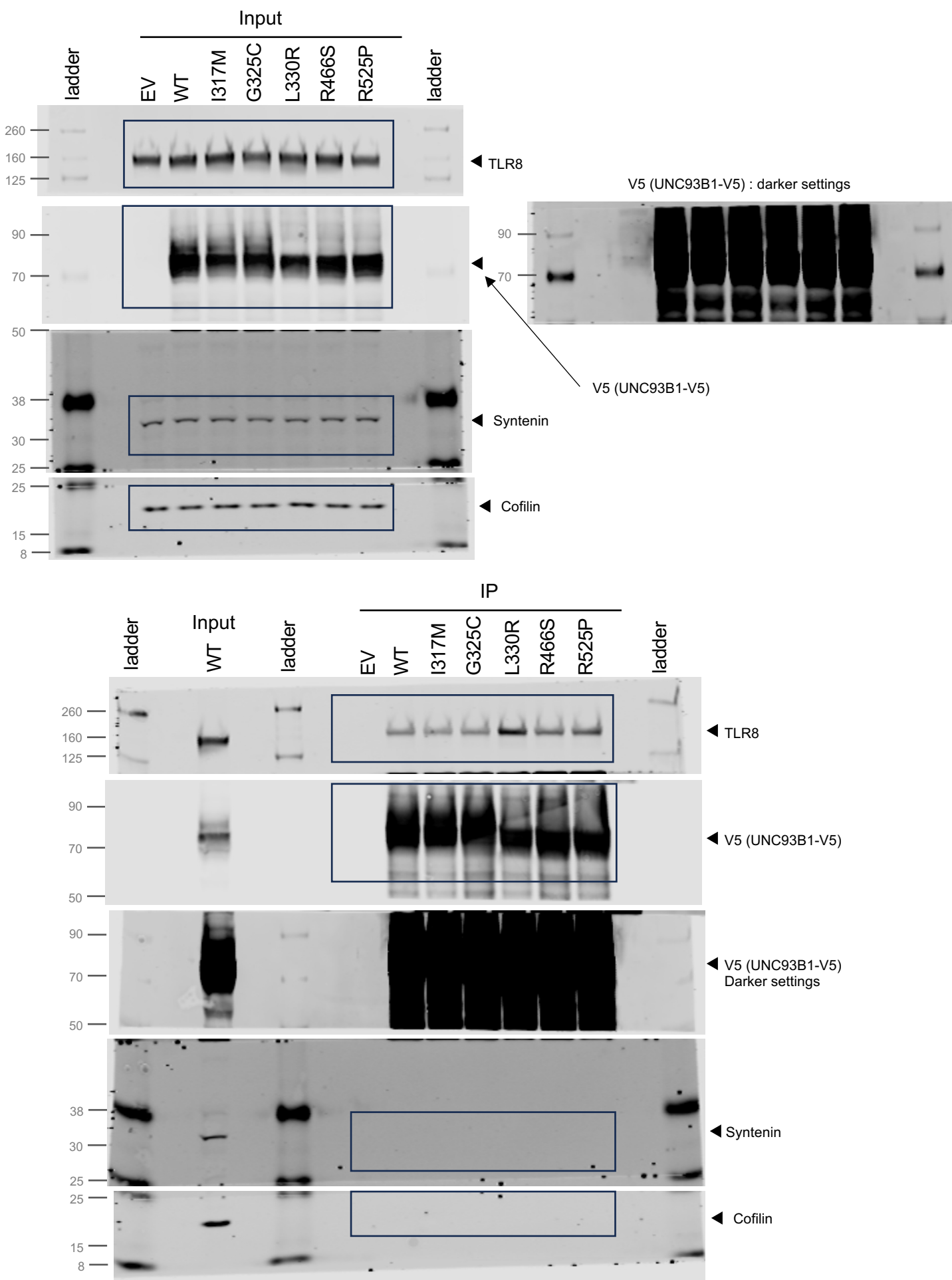

Figure 4I

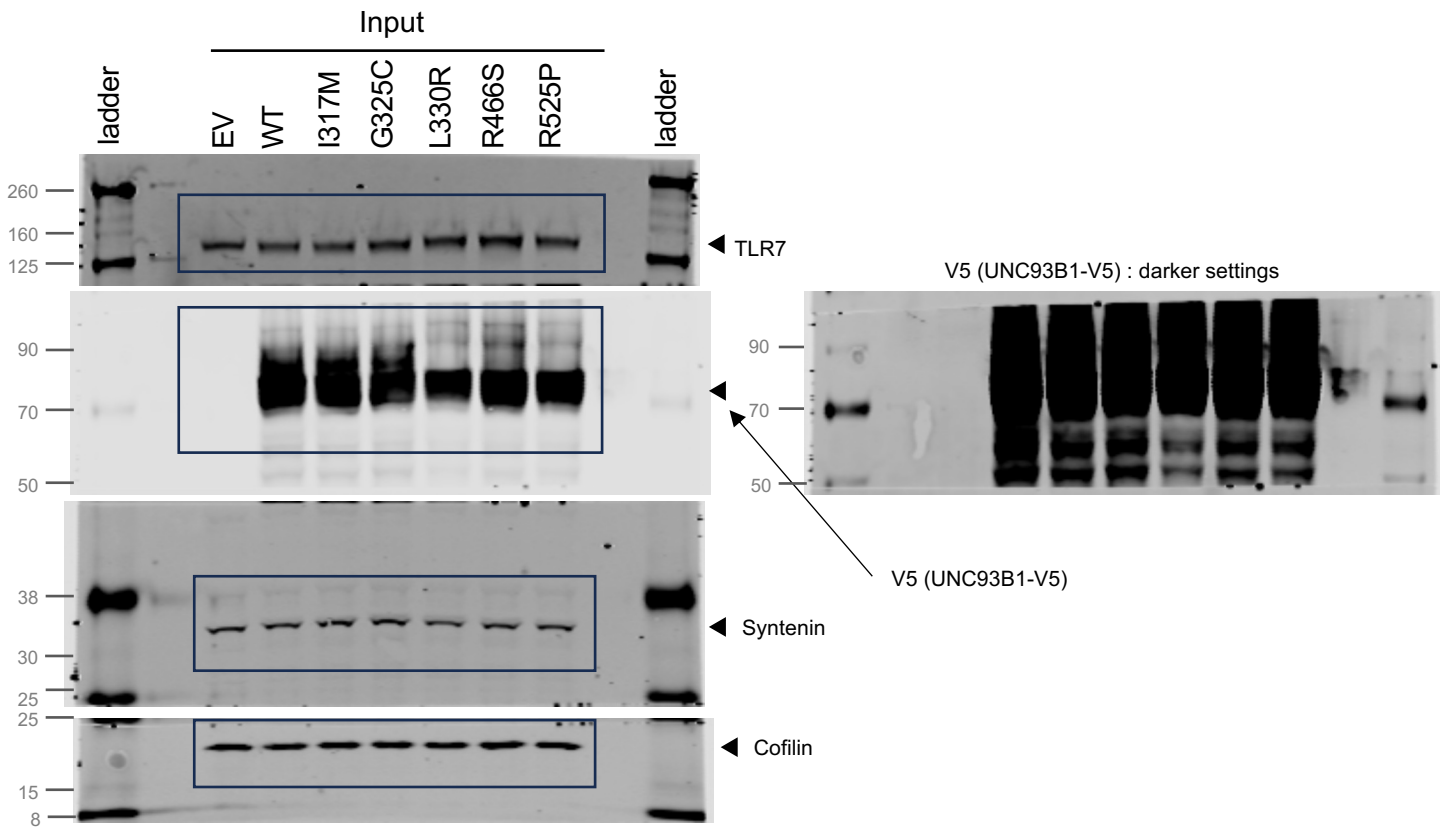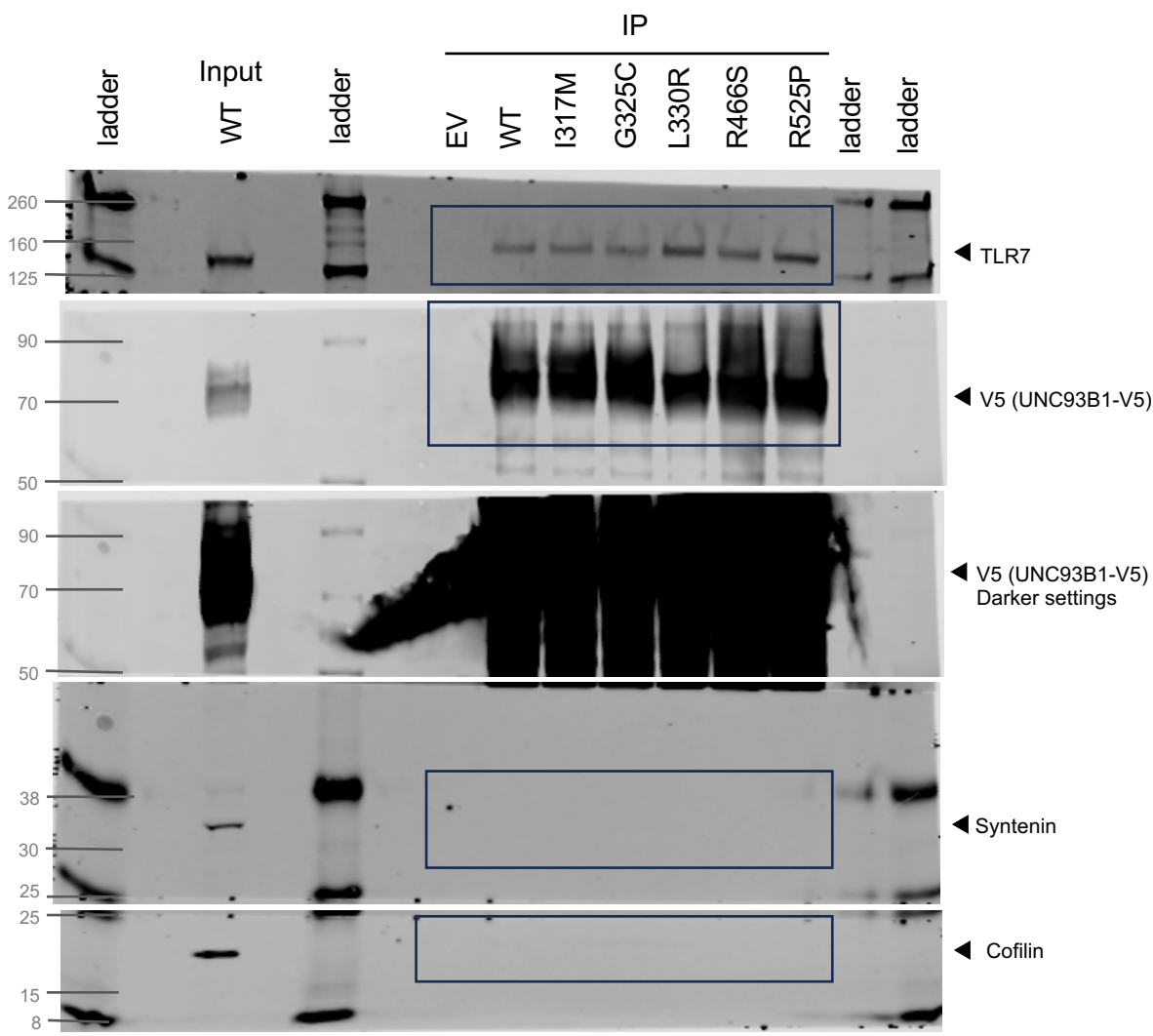

Supplement: SourceData F4 — contains original blots for Fig. 4. [file JEM_20232066_SourceDataF4.pdf]
